# Discrimination of Single-Nucleotide Variants Based on an Allele-Specific Hybridization Chain Reaction and Smartphone Detection

**DOI:** 10.1021/acssensors.1c02220

**Published:** 2022-02-21

**Authors:** Ana Lázaro, Ángel Maquieira, Luis A. Tortajada-Genaro

**Affiliations:** † Instituto Interuniversitario de Investigación de Reconocimiento Molecular y Desarrollo Tecnológico (IDM), Universitat Politècnica de València, Universitat de València, Camino de Vera s/n, 46022 Valencia, Spain; ‡ Unidad Mixta UPV-La Fe, Nanomedicine and Sensors, Av. Fernando Abril Martorell, 46026 Valencia, Spain

**Keywords:** DNA biosensing, single-nucleotide mutation, allele-specific probe, hybridization chain reaction, cancer biomarker genes

## Abstract

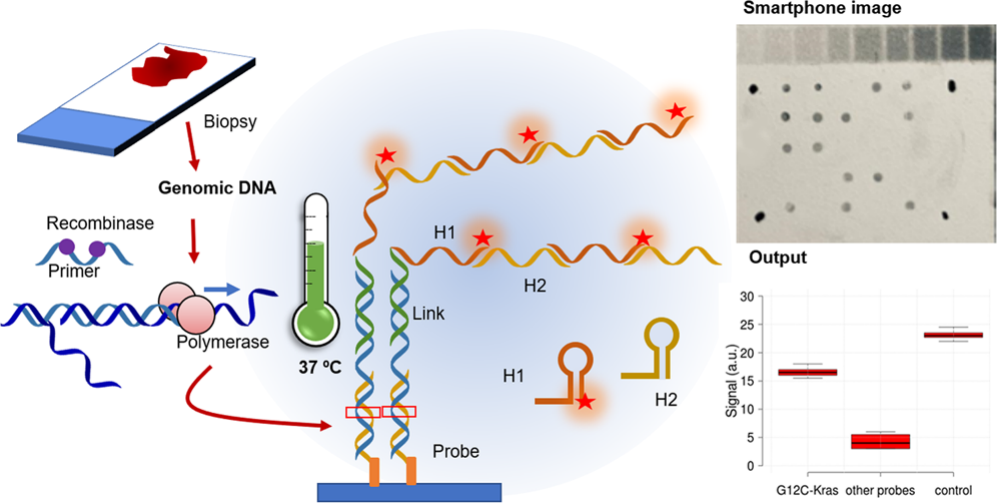

Massive DNA testing requires novel technologies to support a sustainable health system. In recent years, DNA superstructures have emerged as alternative probes and transducers. We, herein, report a multiplexed and highly sensitive approach based on an allele-specific hybridization chain reaction (AS-HCR) in the array format to detect single-nucleotide variants. Fast isothermal amplification was developed before activating the HCR process on a chip to work with genomic DNA. The assay principle was demonstrated, and the variables for integrating the AS-HCR process and smartphone-based detection were also studied. The results were compared to a conventional polymerase reaction chain (PCR)-based test. The developed multiplex method enabled higher selectivity against single-base mismatch sequences at concentrations as low as 10^3^ copies with a limit of detection of 0.7% of the mutant DNA percentage and good reproducibility (relative error: 5% for intra-assay and 17% for interassay). As proof of concept, the AS-HCR method was applied to clinical samples, including human cell cultures and biopsied tissues of cancer patients. Accurate identification of single-nucleotide mutations in *KRAS* and *NRAS* genes was validated, considering those obtained from the reference sequencing method. To conclude, AS-HCR is a rapid, simple, accurate, and cost-effective isothermal method that detects clinically relevant genetic variants and has a high potential for point-of-care demands.

Certain changes in a specific position in the genome sequence, called single-nucleotide variations (SNV), are closely associated with genetic diseases and cancer.^1^ Therefore, disease-related SNVs serve as biomarkers for efficient clinical diagnosis and prognosis based on the genomic profile of primary tumors.^2^ However, the genotyping of SNVs is difficult due to close molecular similarity and their presence at trace levels.^3^ A specific copy number increase is required to detect and avoid inference from high-abundant variants.^4^ The most widely used amplification methods are thermocycling techniques, including a polymerase reaction chain (PCR)^5^ and a ligase chain reaction.^6^ Despite their good performance, the requirement of sophisticated equipment considerably limits applications for point-of-care testing (POC).^7^ Important signs of progress have been achieved in the last years, developing biosensing methods^8−10^ and microfluidics-integrated sensors^11^ with excellent single-base specificity.

Isothermal techniques are appealing alternative tools that work with simple assays and avoid heating/cooling cycles. The first category is enzyme-based methods, such as loop-mediated amplification (LAMP), helicase-dependent amplification (HDA), and recombinase polymerase amplification (RPA).^12^ For instance, their direct integration into consumer electronic devices has allowed the diagnosis of diseases in resource-limited settings.^13,14^ Another innovative approach is to use isothermal reactions as a previous step in recognition/detection assays. Here, the goal is to achieve the required sensitivity based on the generation of small-sized products that improve access to probes^15^ or the biorecognition process, such as nucleic acid detection systems supported on clustered regularly interspaced short palindromic repeats (CRISPR/Cas).^16^

The second group of isothermal techniques is enzyme-free methods, such as toehold-mediated strand displacement amplification^17^ and a hybridization chain reaction (HCR).^18^ In a typical HCR approach, recognizing a target initiates the cross-opening of two DNA hairpins to yield a DNA superstructure of nicked amplified double helices capable of flexible detection.^19^ Due to their simplicity and excellent efficiency, HCRs have been combined with different molecular reporters and readout detection approaches, enabling the sensitive detection of certain nucleic acids.^20,21^ Nevertheless, HCR-based methods remain in the laboratory stage, and their practical applications are still challenging.^22^ To date, there have been two critical limitations: (i) given the structural complexity of genomic DNA (gDNA), only short DNA sequences from clinical samples are amplified by HCR, such as microRNAs,^23^ short gene sequences,^24^ and specific circulating tumor DNA^25^ and (ii) none of the reported studies identify the change in a single nucleotide in gDNA due to the hybridization complexity associated with secondary structures of nucleic acids.^26^

The discrimination of multiple SNVs in gDNA is herein presented. The assay principle consisted of an allele-specific hybridization chain reaction in the array format (AS-HCR), including a previous short RPA. Thus, the double isothermal amplification process is sensitive enough even for a few target copies. By focusing on supporting personalized medicine in low-resource settings, a simple, reliable colorimetric detection system for POC is also developed. A smartphone was selected for its widespread presence, portability, and capacity to transmit data at a user-friendly interface as a biomedical reader.^27^ The study also included the analysis of method requirements and its versatility to identify other important SNV in critical genes for developing diseases.

## Results and Discussion

### Principle of the Genotyping of SNVs Based on AS-HCR

**Figure 1:**
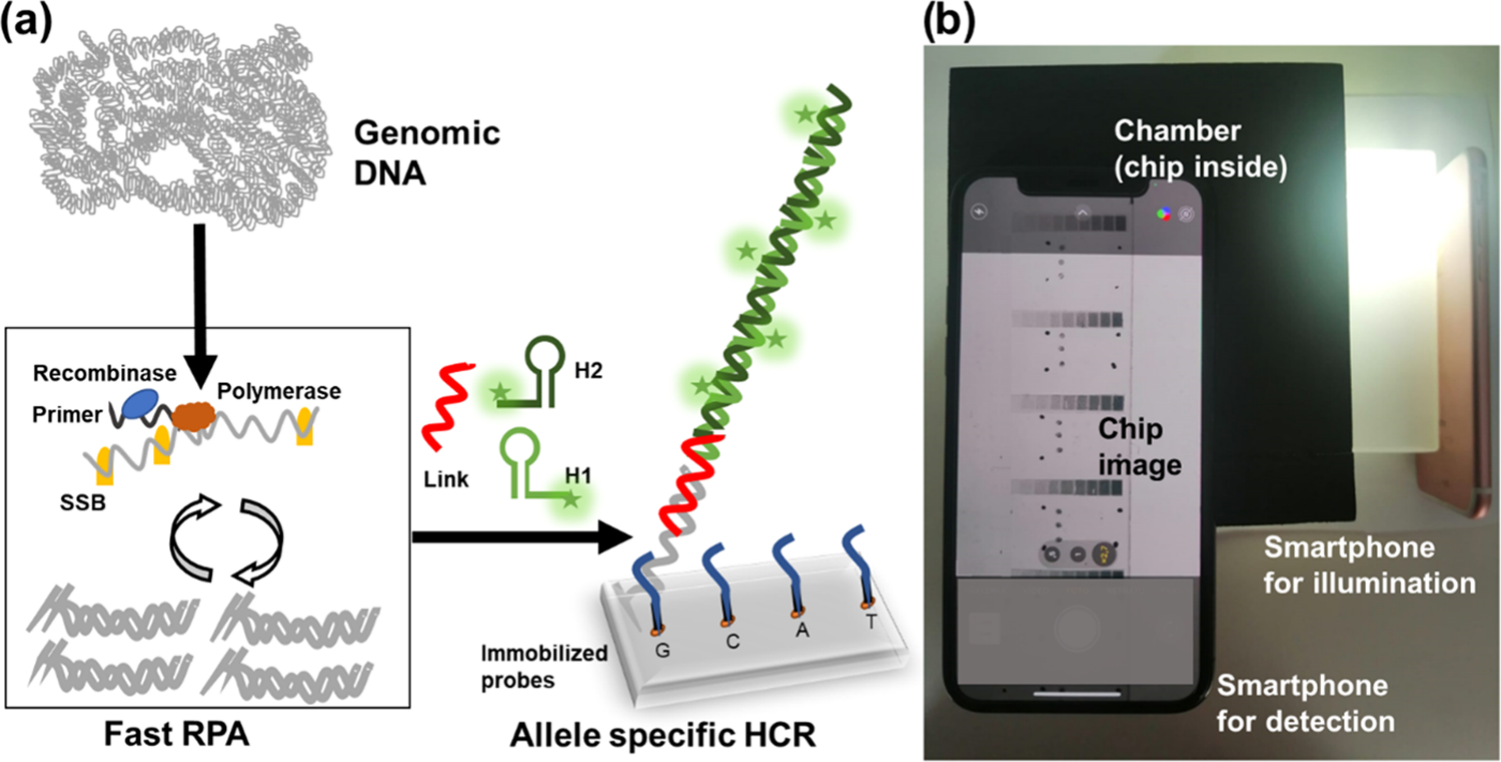
Scheme of genotyping by AS-HCR with colorimetric detection. (a) Mechanism of the AS-HCR method for identifying single-nucleotide variants from genomic DNA and (b) assembly for smartphone detection.

The schematic of genotyping by AS-HCR is shown in Figure 1a. The strategy is based on two isothermal recognition events between specific oligonucleotides (primers, probes, and reporters) and a target sequence. First, the specific human genome region is amplified by a fast isothermal technique, recombinase polymerase amplification (RPA), to generate short-length products. Subsequently, selective sequential hybridizations are activated on the chip surface. The target DNA sequence (G, C, A, or T variant) hybridizes with the corresponding immobilized allele-specific probe. Then, the 5′-end of the product hybridizes to the link sequence. At the same time, the link acts as a universal initiator to trigger the cascade self-assembly of the two partially complementary DNA hairpins (H1 and H2). The labeling of hairpins yields a hybridization pattern by discriminating SNVs. The approach is compatible with several transducer methods, such as optical or electrochemical. The presented study-developed colorimetric chip staining was based on antigen/antibody recognition and metallographic development. Thus, chip imaging can be obtained by a smartphone.

### HCR Method Setup for Short Templates and the Human Genome

**Figure 2:**
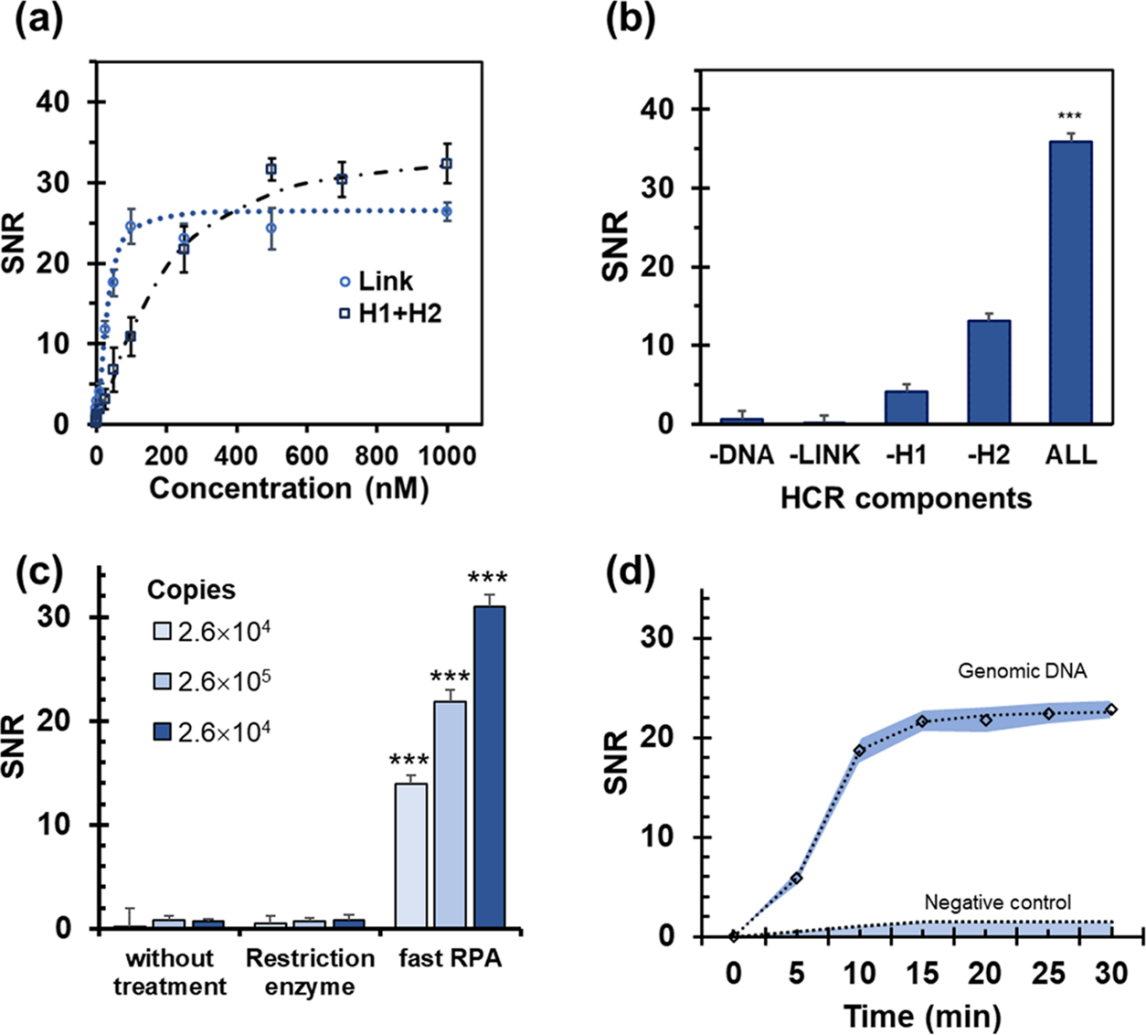
HCR method setup. (a) Essential components for HCR (the – sign indicates the component that was not added for each reaction). (b) Concentration of link ([H1] = [H2] = 500 nM) and H1 plus H2 ([Link] = 500 nM). (c) Comparison of the detected signals depending on sample treatment. (d) Effect of RPA time. Target: *KRAS* gene (codons 12-13). *** Student’s *t*-test *p*-values < 0.05. Sample: human DNA.

Solid-phase hybridization assays in an array format and later optical detection were studied on plastic chips. Conventional HCR reaction requirements were examined using a 72-nucleotide sequence as a model template. The study of the oligonucleotide design (Tables S1–S3, Figures S1 and S2) and experimental variables (Figures S3 and S4) yielded high-intensity signals. The most relevant variable in the amplification performance was the sequences and concentrations of link and H1/H2 oligonucleotides (Figure 2a). The selectivity of assays was confirmed because only when all of the components were present, a long polymer structure of dsDNA was formed and easily detected (SNR > 30) (Figure 2b). These observations agreed with the experimental conditions described in previous studies.^28^ Applied to short templates, HCR proved to be a powerful enzyme-free amplification technique, and the stoichiometric ratio between the target molecule and markers substantially increased (8.8-fold). However, the method failed to directly use the gDNA (6.4 billion base pairs) extracted from human cells as a template. From gDNA, steric hindrances and strand template stability prevented the formation of the link/template complex, and HCR could not be activated regardless of the initial concentration.

To improve its applicability in actual clinical practice, gDNA treatment was studied before the HCR method (Figure 2c). The first option was to use restriction enzymes for genome fragmentation. Although DNA digestion was achieved, there were no positive signals. We hypothesized that the presence of steric hindrances still affected recognition processes.^29^

The second option was to incorporate an isothermal preamplification step. RPA was selected for its high yield, low working temperature, and high reaction rate. Although the two techniques (RPA-HCR) have not been combined to date, the strategy provided the expected results. The formation of a few copies was enough to produce a perfect-match hybrid with the immobilized probe to be recognized for the linker and, consequently, to trigger the HCR reaction (Figure 2d).

We observed that one crucial requisite for successful detection was a correct primer design to yield short amplification products. The estimation involved a competitive hybridization template/link requiring products lower than 80 bp. Another relevant result was that a short amplification step (10 min) sufficed to register detectable signals. Hence, the combination of two highly selective efficient amplification processes (RPA-HCR) led to a fast and straightforward isothermal approach, i.e., compatible with POC applications.

### Smartphone-Based Detection of HCR Products

The detection of the previously described products in an array format was achieved with conventional laboratory instruments such as a chip scanner. The next step was to adjust the method to perform colorimetric smartphone detection for POC applications. To that end, of the different techniques currently available for labeling DNA chips, the immunoassay/nanoparticle system was chosen for its simple protocol.^30^ The setup of smartphone-based detection is described in the Supplementary Information (Figures S5 and S6). Array quality was confirmed by studying the morphology of spots, including cross-sectional profiles, intensity, and diameter homogeneity (Figure S7). Under the selected conditions, each array spot (diameter 250 μm) corresponded to 314 pixels. Positive tests depicted up to SNR = 30 in 16-bit grayscale units, while negative ones fell within the chip background range.

**Figure 3:**
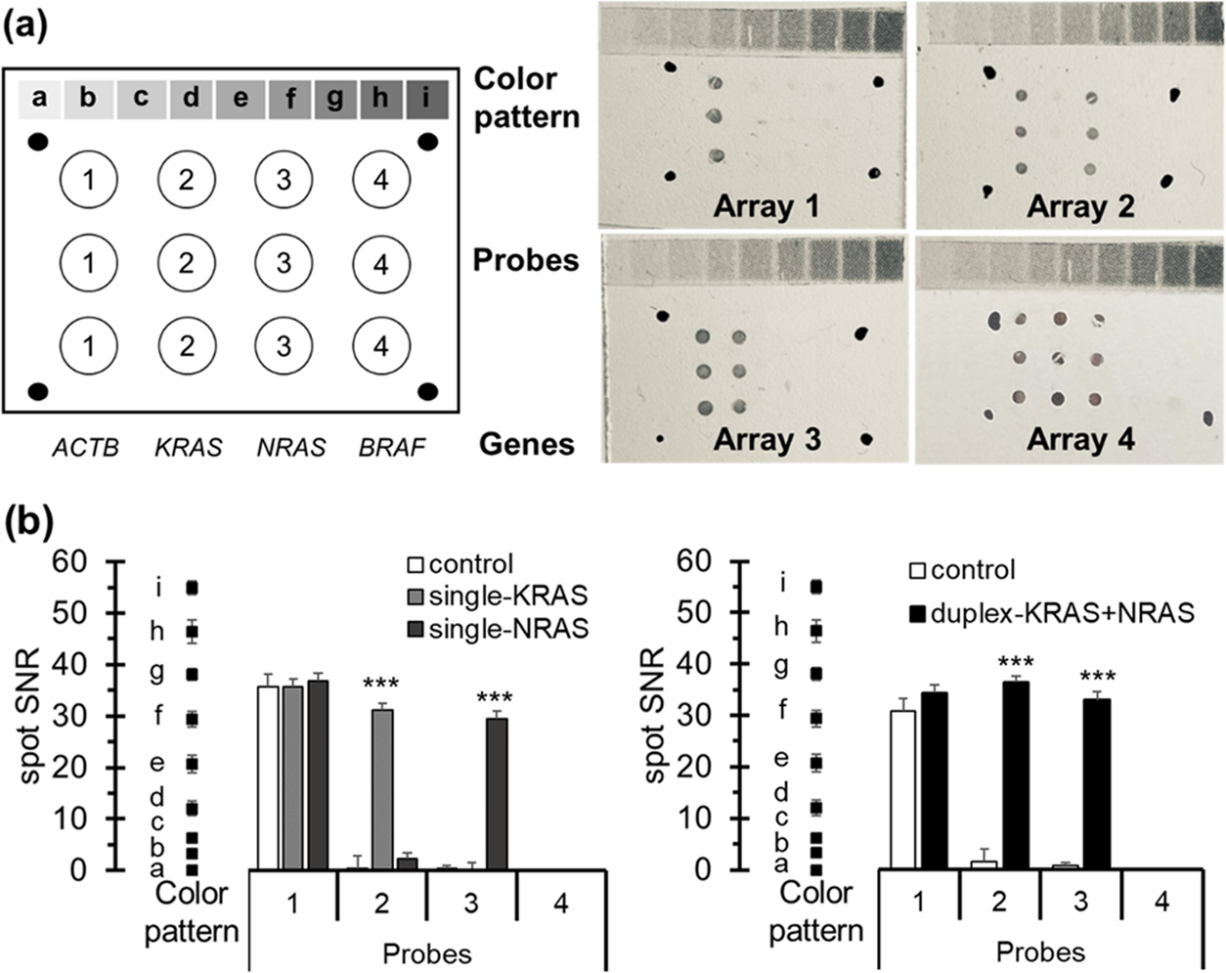
Smartphone detection of RPA-HCR products for the *KRAS* and *NRAS* targets. (a) Qualitative results: layout (left) and resulting images (right) taken by the smartphone using control (array 1), *KRAS* product (array 2), *NRAS* product (array 3), and a mixture of both products (1:1) (array 4). (b) Quantitative results: spot signals for single assays (left) and duplex (right). SNR: signal-to-noise ratio. *** Student’s *t*-test *p*-values < 0.05. The color pattern ranges from *a* (lower intensity) to *i* (higher intensity).

A correct pattern was obtained in the single assay format, as positive responses were registered only for complementary probes of the target RPA products (Figure S8). The same experiments were successfully performed using a mixture of products from two target regions (Figure 3). Qualitative and quantitative responses supported that the assay was feasible for different targets during single and duplex detection. Also, interassay robustness was assured by doing replicates (ANOVA, *p*-value < 0.05). As a result of excellent performance, this study demonstrates the colorimetric smartphone detection of HCR products for the first time. It opens up a new path for developing fast, portable, and easy-to-handle platforms, particularly useful in decentralized scenarios and low-resource health systems.

### Allele-Specific HCR Setup

Previous HCR-based methods can detect DNA sequences but they cannot identify any change of the involved nucleotide.^31^ The challenge of genotyping SNV was approached by considering the integration of AS-HCR and allele-specific probes immobilized on chips in a microarray format.

Specific probes were designed to maximize the selective recognition process for a single nucleotide by considering thermodynamic calculations (Table S1). The results indicated that differences in free energy variation must be significant enough for a discriminatory interaction, with an approximate threshold of 4 kcal/mol, equivalent to an increase in the melting temperature of 6 °C. Moreover, the relative position of probes and the linker was optimized because competition will disrupt the recognition/amplification process if both overlap the same template sequence. The estimation was a separation of nine nucleotides between the probe (3′-end of the target) and the linker (5′-end of the target).

**Figure 4:**
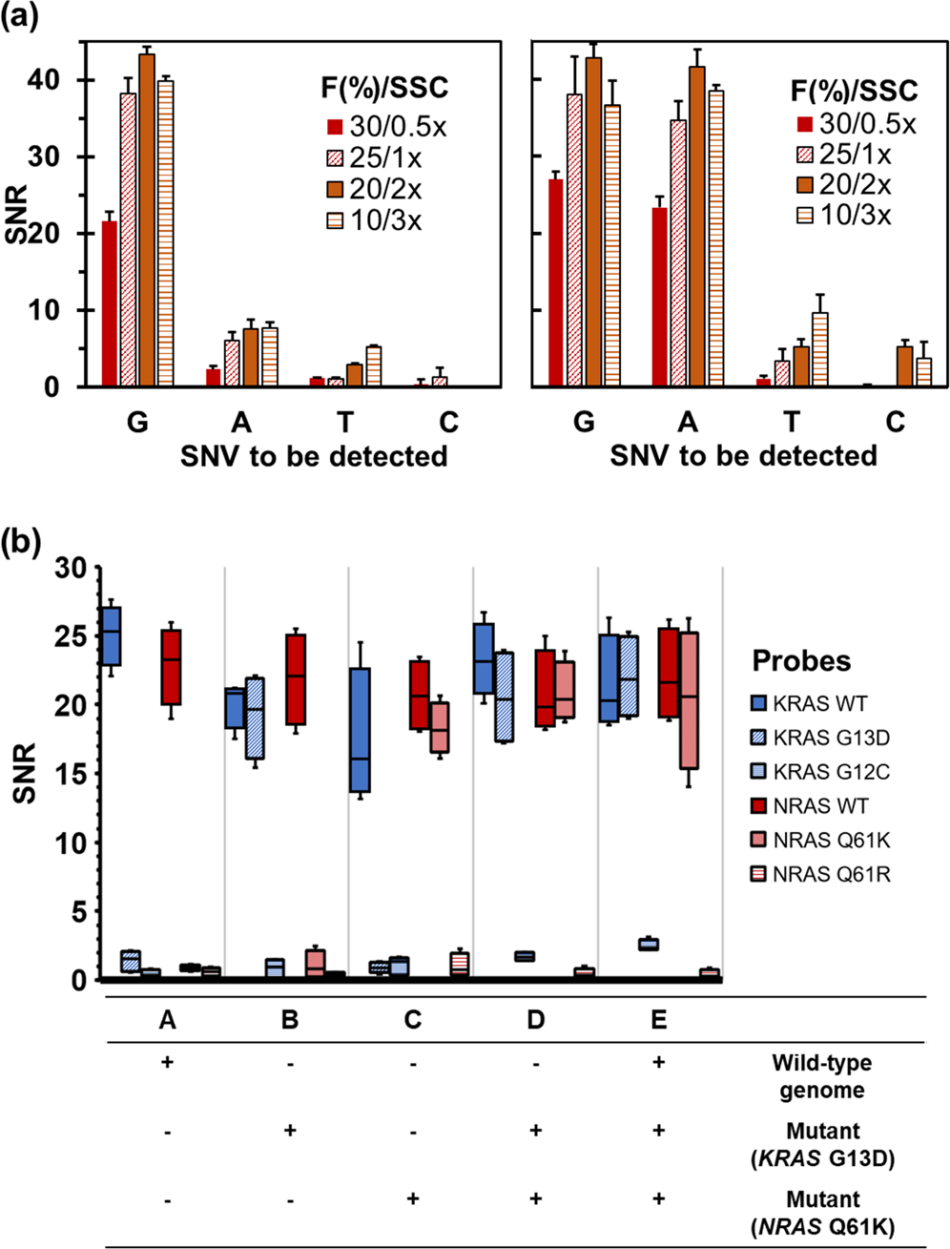
(a) SNV discrimination ability of the wild-type cell line (left) and the mutant cell line (c.38G>A KRAS) (right). F(%): formamide percentage and SSC: sodium saline citrate buffer. (b) Multiplexing ability of AS-HCR using different mixtures of genomic DNA from cell lines (NGS genotyped samples). SNR: signal-to-noise ratio. As the mutant cell lines were heterozygous, wild-type probes also showed a positive signal.

The reaction conditions were revised to maximize hybridization efficiency. The selection criteria were the best discrimination capability when comparing the detection signals from the perfect-match probe and the mismatches addressing the same locus. Regarding the hybridization buffer, the biorecognition process of SNVs was improved by controlling the ionic strength and the concentration of formamide (Figure 4a,b). As expected, reagents stabilized single-stranded DNA by promoting only the entirely complimentary sequences to hybridize.^30^ The highest selectivity with no loss of analytical signal was obtained for 25% formamide and 500 mM citrate buffer (pH = 7). The best responses were obtained by incubating at 37 °C for 30 min.

The correct identification of the present DNA variant was achieved because the spot SNR was 23–42 for the perfect-match probes and 0–9 for the mismatch probes (single-nucleotide change). Consequently, the signal-generating assay was highly specific. For instance, the wild-type cells for the *KRAS* gene only provided positive responses for the correct probe (G probe) with minimal nonspecific intensity in the remaining probe, while the mutant cells (c.38G>A) hybridized to two probes (G and A probes) with similar intensities. These values agree with a gDNA from a human cell culture being heterozygous for this locus.

Therefore, the results demonstrated selective polymer formation by the perfect-match interactions among the corresponding specific probe, the template, the link, and H1/H2. Hence, the accurate discrimination of only one mismatch in one same locus is possible by the AS-HCR method.

### Multiplexed AS-HCR

The next challenge was the simultaneous discrimination of several polymorphisms in a single assay. Multiplexed detection required compatible RPA primers, HCR links, and allele-specific probes to be selected for all of the target SNVs. As proof of concept, duplex-fast RPA for two human genes was optimized and performed from culture cells (SK-N-AS and HCT116). The formed products were hybridized to the probes immobilized in an array format to perform the AS-HCR method (Figure 4c). The recognition profiles corresponded precisely with the expected perfect-match complexes (probe-template) regardless of assay multiplexing. The results depicted that this methodology is a promising tool to identify the SNVs from gDNA accurately. Thus, RPA combined with AS-HCR in the microarray format can perform the multiplex discrimination of different loci and simultaneously determine many samples by simplifying the analysis and reducing times compared to other detection techniques.

### Comparison to the PCR-Based Array and Other DNA Biosensors

**Figure 5:**
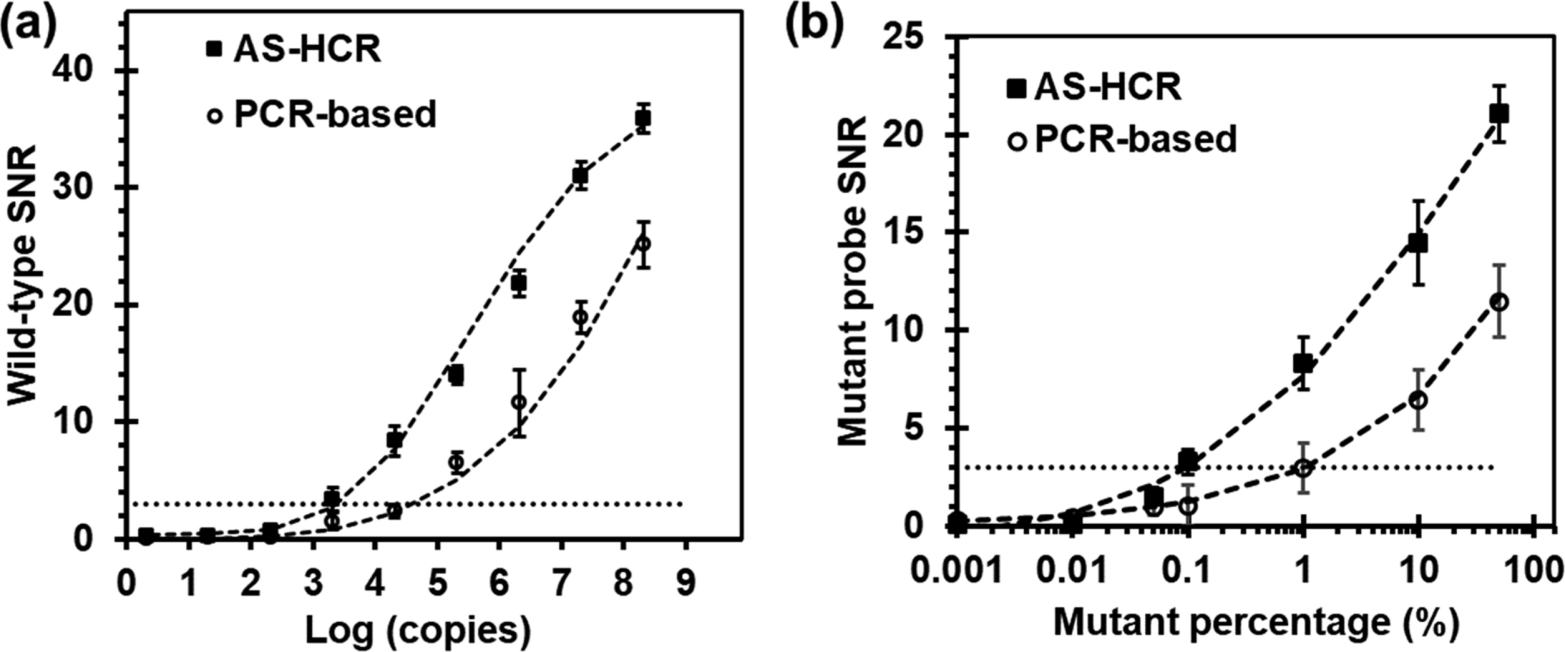
Comparison of the assay sensitivity of the AS-HCR and PCR-based method. (a) Effect of the DNA copy number and (b) effect of the mutant template percentage, where the total amount remained constant. SNR: signal-to-noise ratio. Mutant: *KRAS* c.34G>T.

A conventional allele-specific method based on PCR and a hybridization chip was chosen to evaluate the biosensing performances of AS-HCR (Table S4). Sensitivity was obtained from serial dilutions of the human genomic template and gDNA mixtures. As shown in Figure 5, an exponential correlation was observed between the spot response and template copies. However, the copies required for the novel method were 1.7-fold lower than the PCR approach. The detection limits, expressed in mutant abundance, were 1.1% for the PCR-based method and 0.2% for AS-HCR, i.e., almost fivefold lower. Each copy of the initiator could trigger the HCR event, which resulted in the linkage of many oligonucleotides and showed very high potential in signal amplification for DNA detection purposes.

Reproducibility was determined from the replicated assays and expressed as a relative standard deviation. Values were 5% for intra-assay and 17% for interassay and, thus, the high consistency among the parallel results confirmed the robustness. Therefore, AS-HCR allowed the specific DNA variants to be identified despite the high wild-type/mutant cell ratio.

Concerning the operational features, the first difference lay in the analysis time, which was 1 h for the novel method (amplification: 10 min, chip assay: 50 min) and 2 h for the conventional method (amplification: 70 min, chip assay: 50 min). Second, AS-HCR was isothermal, low cost, and instrument-free. Third, this method was less sensitive to the inhibiting factors of the PCR amplification often found in complex matrices.^32^ Finally, some authors have pointed out that HCR approaches can effectively reduce false-positive results and cross-contamination from amplicons or carry-over pollution, which frequently occur in PCR.^20^ The reason is that the repetitive copy of the template is not controlled.

Although further research is needed, our method depicted some unique advantages over sensing processes in traditional DNA assays, approaching the requirements for an ASSURED test. The use of cheap chips, reagents, and a smartphone as an array reader supports DNA testing in low-resource laboratories (affordable). Most conventional biosensors rely on the hybridization between target molecules and signal probes at a 1:1 stoichiometric ratio, which restricts detection sensitivity.^33^ In this novel approach, a single target DNA molecule produces a cascade of hybridizations to form a long concatemers structure and, thus, generates a one-to-multiple amplification effect (sensitive). AS-HCR is an isothermal alternative that does not involve a thermal cycler (equipment-free). Moreover, the specific recognition process (selective and robust) is compatible with several transduction principles depending on the hairpin marker. If the two hairpins are labeled with digoxigenin, an easy immunoassay can be performed, and the resulting arrays can be read by the colorimetric mode or naked-eye detection (user-friendly). Finally, it can be used for POC applications, thanks to its simplicity, portability, and low cost in both acquisition and maintenance terms (deliverable to end-users).

### Genotyping Patient Samples

**Figure 6:**
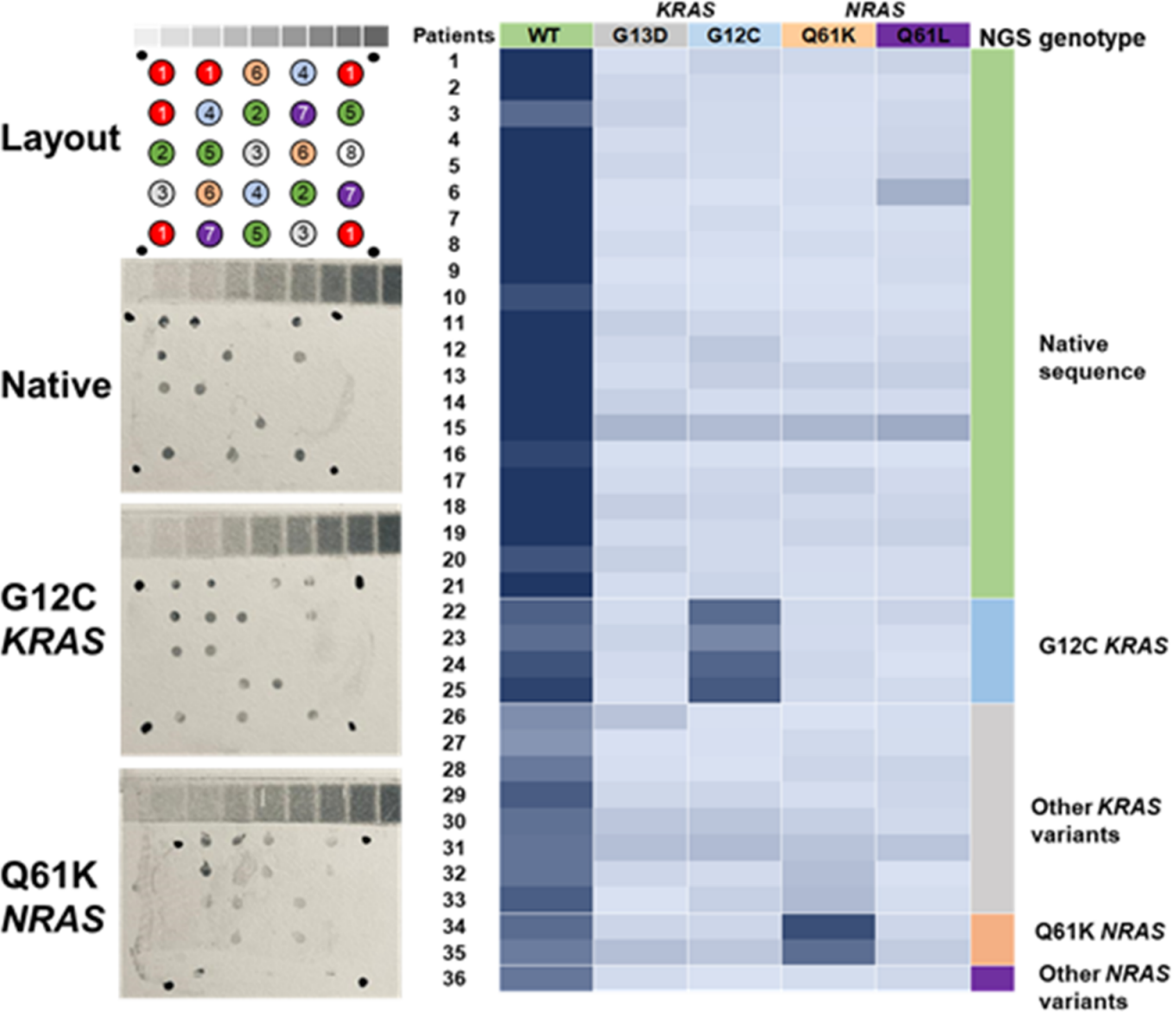
Identification of specific mutations from cancer patients using the AS-HCR method. (Left) Spot layout and images obtained by a smartphone. (Right) Heating map of probe responses, classified as the population groups identified by AS-HCR and ion torrent sequencing technology. Probes: (1) positive control, (2) WT-KRAS, (3) G13D-KRAS, (4) G12C-KRAS, (5) WT-NRAS, (6) Q61K-NRAS, (7) Q61R-NRAS, and (8) negative control.

The biosensing ability to distinguish SNVs in real samples, related to solid cancer screening, was tested. As proof of concept, the proposed method was applied to identify the specific mutations in the *KRAS* and *NRAS* genes from biopsied tumors tissues, which were formalin-fixed and paraffin-embedded (FFPE). The validation set was obtained from patients with metastatic colon cancer (*n* = 36). The analysis was conducted as a blind test (Figures 6a and S8).

In all cases, the high sensitivity gave a positive response (SNR > 3) for the probes corresponding to the wild-type genotype. However, in some patients, a small amount of and/or poor-quality DNA is due to the nature of clinical samples and their conservation mode. According to the hybridization patterns, positive signals were associated with the p.G12C mutation (c.34G>T) in the *KRAS* gene and the p.Q61K mutation (c.181C>A) in the *NRAS* gene. Four (11.11%) and two patients (5.56%) were, respectively, identified. Negligible signals were obtained for the other probes.

Assay accuracy was validated by the independent sequencing of patient samples (Figure 6b and Table S5). Complete agreement was obtained, which demonstrates the capability of AS-HCR to identify specific mutations independently of the mutation type and its position. In addition, the small proportion of mutated cells in tumor tissue did not limit the assay’s success.

Although further research is needed, the developed approach is adequate for clinical applications. The AS-HCR method provides key genetic information that can be used to apply personalized medicine to patients with metastatic colon cancer. Considering the state-of-art (Tables S6 and S7), our approach can be classified as a high-moderate sensitive method for mutational analyses (0.1–1%) useful for solid tissues. This method is much simpler and cheaper than instrumental methods and can be applied to other diseases that require genomic screening, focused on the genotyping of nucleotide variations.^6,33,34^ These findings facilitate the differential diagnosis and determination of disease prognosis, as well as identification of the drug resistance of tumors and, hence, therapy selection.^2,35,36^Table S8 shows more examples of the possible applications of the novel technology based on the discrimination of nucleotide variations.

## Conclusions

Currently, reliable, fast, economical, and sensitive methods to discriminate SNVs for clinical applications and other scientific fields need to be developed. The developed HCR method is a powerful, cost-effective alternative for this purpose. In this study, we made several relevant research advances: (i) the treatment of the gDNA with RPA allowed an HCR approach to be applied to complex clinical samples; (ii) the integration of alleles specific to hybridization chain reactions provides the ultrasensitive and selective genotyping of several SNVs; and (iii) as the link acts as the trigger for the HCR reaction, the multiplexing capability is high because the amplification is based on a universal pair of oligonucleotides (H1 and H2). Thus, it is possible to extend this technology to other targets by designing the correct probe and link. (iv) Consumer electronic devices, such as smartphones, simplify the implementation of molecular methods outside centralized laboratories and (v) the approach shows a high-throughput analysis, portability, and availability. Given its advantages, this novel method implies promising DNA testing opportunities by supporting personalized medicine. The massive genotyping of specific biomarkers (target SNVs) will improve the diagnosis, prognosis, and assignment of the appropriate treatment to each individual patient.

## Methods

### Reagents

The fabrication of chips is described in the Supporting Information. The hybridization buffer was sodium citrate 45 mM, NaCl 450 mM, 25% formamide, and 2.5× Denhardt’s reagent, adjusted to pH 7. The positive control of allele-specific hybridization (digoxigenin-labeled amplification product of the β-actin gene) was added (1 nM). Washing buffers were dilutions of saline citrate buffer (1:10 and 1:100).

The studied application was the discrimination of SNVs in oncogenes. A set of oligonucleotides (primers, probe, and link) were complementary to a specific region close to each target SNV (Figures SI1 and SI2). In this study, the targets were the point mutations located at *KRAS* (codons 12 and 13) and *NRAS* (codon 61) genes. The design criteria and the list of the used oligonucleotides, supplied by Eurofins (Luxembourg), are shown in Tables SI1 and SI2.

### Patients

Human SK-N-AS cells (brain) with wild-type *KRAS* and HCT116 cells with mutant *KRAS* G13D (colon cancer) were purchased from the American Type Culture Collection (ATCC) and used for method optimization purposes (passage number lower than 4).

The pathologically confirmed metastatic colorectal cancer DNA samples (*n* = 36) were obtained from formalin-fixed paraffin-embedded (FFPE) biopsy tissues of patients from the Oncologic Service of the Hospital Clínico Universitario La Fe (Valencia, Spain). Informed consent was provided by each patient. Genomic DNA was extracted as described in the Supporting Information.

### RPA

Fast isothermal amplification was performed using the TwistAmp Basic RPA kit (TwistDx, U.K.). Reaction mixtures (12.5 μL) were prepared with rehydrated buffer, 14 mM magnesium acetate, 200 nM each forward and reverse primer, 4 ng of gDNA, and the enzyme pellet. Solutions were incubated at 37 °C for 10 min in an oven (Beschickung—Loading Model 100-800, Germany).

### AS-HCR Process

RPA products (6 μL) were mixed with the hybridization buffer (20 μL) and the oligonucleotide solution (4 μL), with final concentrations of 100 nM of links and 500 nM of H1 and H2. Solutions were denatured (92 °C, 10 min) for opening the double-strand RPA product and transferred to the chip of 12-assay zones. The chip was incubated in an oven at 37 °C for 30 min and rinsed once with washing buffer for 1 min.

### Detection

To detect the complex formed by the HCR reaction, the chip was stained as described previously.^24^ The imaging system was an iPhone 11 Pro’s triple camera, which offers a ×2.7 optical zoom, a 12 MP resolution, and an f1.8 aperture in a specific reading assembly (Figure 1b). After sending the images to a PC, Image J free software was used to convert images into a tagged image file format on a 16-bit grayscale and analyze them. The analytical signals were considered the variation of the spot intensity and the chip background. Signal-to-noise ratios (SNR) were obtained by dividing the mean spot signals between the associated noise values, calculated as the standard deviation from 15 blank measurements. The limit of detection limits was inferred from the experimental concentration corresponding to the SNR equal to 3.

### Reference Methods

PCR combining with allele-specific hybridization was used to compare the analytical performance of AS-HCR using a smartphone and a chip scanner. Ion Torrent PGM technology (ThermoFisher Scientific) was applied to validate the somatic mutations detection of patient samples. Both methods are described in the Supporting Information.

## Supporting Information Available

Experimental data including the design of oligonucleotides, HCR method setup, smartphone-based detection setup, reference methods, and application to clinical samples (PDF)

## Notes

Research involving human subjects complied with all of the relevant national regulations and institutional policies followed the Declaration of Helsinki tenets (as revised in 2013) and was approved by the authors’ Institutional Review Board (Ethics Committee LAFE2015/096). Informed consent was obtained from all of the individuals included in this study.
