# Supplementary material for: Discrimination of Single-Nucleotide Variants Based on an Allele-Specific Hybridization Chain Reaction and Smartphone Detection
Source: ACS Sens. 2022 Feb 21;7(3):758–65. doi: 10.1021/acssensors.1c02220 (PMC8961872; doi:10.1021/acssensors.1c02220)
Supplement: Supplementary file 1 — se1c02220_si_001.pdf [file se1c02220_si_001.pdf]

Discrimination of single nucleotide variants based on allele-specific hybridization chain reaction and smartphone detection

Ana Lázaro,[a] Ángel Maquieira,[a, b] Luis A. Tortajada-Genaro,[a,b,*]
[a] Instituto Interuniversitario de Investigación de Reconocimiento Molecular y Desarrollo Tecnológico (IDM), Universitat Politècnica de València, Universitat de València, Camino de Vera s/n, 46022, Valencia, Spain.
[b] Unidad Mixta UPV-La Fe, Nanomedicine and Sensors, Av. Fernando Abril Martorell, 46026, Valencia, Spain
Email: luitorge@qim.upv.es

<u>**Table of Contents**</u>

1. Design of oligonucleotides
Figure S1. Structure of the H1 and H2 oligonucleotides.
Figure S2. Design of oligonucleotides for the AS-HCR method.
Table S1. Thermodynamic analysis of AS-HCR oligonucleotides.
Table S2. Sequences of the oligonucleotides used for the AS-HCR method.

2. HCR method setup
Figure S3. Evaluation of conventional HCR reaction conditions.
Table S3. Sequences of the oligonucleotides used as biosensing process controls.
Figure S4. Linear vs. HCR approaches.

3. Smartphone-based detection setup
Figure S5. Assembly for the smartphone-based detection of HCR products.
Figure S6. Optimization of chip immunostaining for smartphone-based detection in the colorimetric mode.
Figure S7. Assessment of the array quality obtained by smartphone detection by measuring the cross-section profiles of spots.

4. HCR method from RPA products
Figure S8. HCR method from RPA products

5. Allele-specific HCR
Table S4. Comparison of PCR-chip and RPA-HCR methods.

6. Application to clinical samples
Figure S9. Images obtained from patient samples.
Table S5. Mutation analysis of clinical samples by PCR-based and NGS techniques.
Table S6. Examples of different HCR sensing approaches classified according to the sensing principle.
Table S7. Currently available platforms and closely related SNV strategies for RAS genotyping.

7. References

1. Design of oligonucleotides

Selection of the H1 and H2 oligos. A correct design was crucial for effective amplification because any slight change in hairpins could lead to an error in the HCR process. The thermodynamic analysis of the candidate oligonucleotides (different lengths and %GC) reported critical information about the sequence of H1, H2, and their secondary structure. Hairpin sets had the short loop protected by the long stem to store potential energy. Reducing toehold and loop size promotes hairpin metastability, while increasing it activates hairpin polymerization.[1] The typical structure utilized in HCR systems is a 6-base loop and 18 bp stem, and both H1 and H2 oligos are symmetric.[2,3] In our approach, a shorter stem structure and a pair of sticky ends were chosen to reduce any unexpected polymerization. The structure of the final design of H1 and H2 (38 nucleotides) had 12 base pair stems, 6 base pair loops, and 8 base complementary tails (Figure S1). Both molecules were functionalized with a reporter group in the optical detection mode. The immunostaining option was chosen for the colorimetric approach, and hairpins were modified at their 5' end with digoxigenin as the reporter molecule.

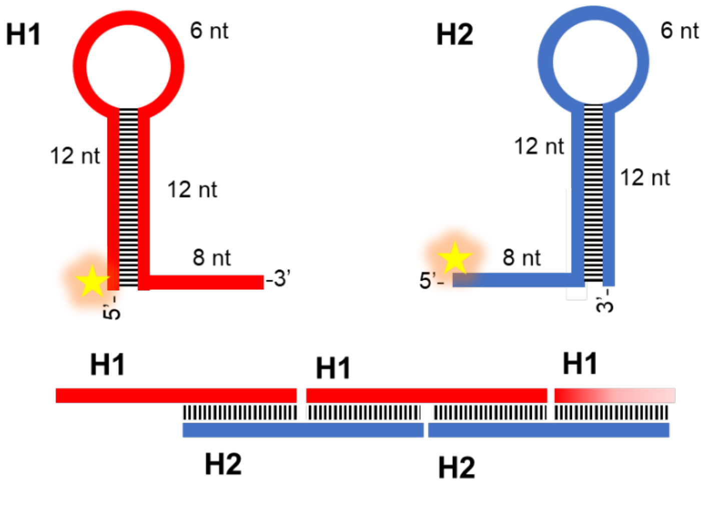

Figure S1. Structure of the H1 and H2 oligonucleotides.

<u>*Target-specific oligonucleotides*</u>. Oligonucleotide sets were designed to analyze the most important hotspot mutations in Kirsten rat sarcoma-2 oncogene (*KRAS,* codons 12 and 13) and Neuroblastoma Ras viral oncogene (*NRAS,* codon 61). Therefore, oligonucleotides were complementary to a specific region close to the target SNVs (Figure S2). Primer sequences defined the region extremes, probe sequences included the target nucleotide at a central position, and a link sequence was designed at one region extreme. The specific sequences were chosen based on the selection criteria described in previous papers for single nucleotide polymorphisms[4,5] and single-point mutations.[6]

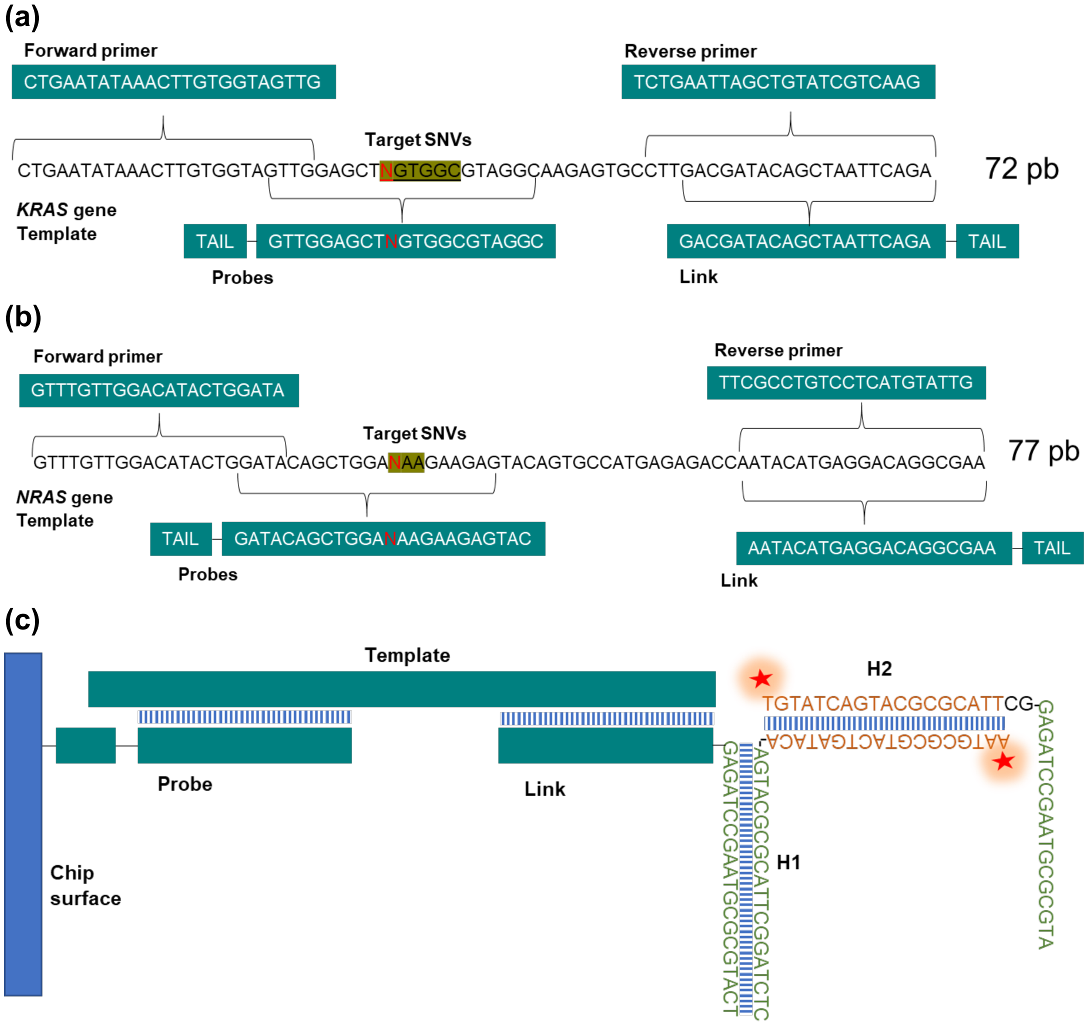

Figure S2. Design of the oligonucleotides for the AS-HCR method: (a) *KRAS* gene (codon 12-13). (b) *NRAS* gene (codon 61). (c) Universal enzyme-free amplification based on HCR.

In order to achieve multiplexing capability, a specific link was designed as an intermediate to trigger a universal HCR reaction by considering the requirement previously described for single assays.[7] For this purpose, the link was designed with a specific secondary structure. It was formed by base-pair stems, loops, and two tails at the 5'-end and the 3'-end. Therefore, the base-pair hybridization of the template/link and link/H1 hybrids was more stable than the hairpin structure of H1 (Table S1). The list of the chosen oligonucleotides is shown in Table S2.

The estimated free energy variation for the complexes between the specific probes to its fully complementary target sequence varied from −22.0 to −26.6 kcal/mol. The values calculated for the mismatched complexes ranged between −17.4 and −22.7 kcal/mol. The estimated differences were meaningful enough for a discriminatory assay, and about 4.8 kcal/mol was the equivalent to an increment in the melting temperature of 6.6 ºC.

Table S1. Thermodynamic analysis of the AS-HCR oligonucleotides. (a) Chip hybridization (b) Link/H1/H2 system Software: Dinamelt software for secondary structures.[8]

(a)	Oligo	Sequence (5'-3')	ΔG Perfect-match template	mean ΔG Mismatch templates	ΔG Difference
KRAS gene	Probe 1	GTTGGAGCTGGTGGCGTAGGC	-25.4	-22.7	2.7
	Probe 2	AGTTGGAGCTGTTGGCGTAGGC	-25.5	-20.8	4.7
	Probe 3	AGTTGGAGCTGATGGCGTAGGC	-25.3	-21.0	4.3
	Probe 4	AGTTGGAGCTGCTGGCGTAGGC	-26.6	-20.2	6.4
	Probe 5	GTTGGAGCTGGTGACGTAGGC	-24.0	-18.2	5.8
NRAS gene	Probe 1	ATACAGCTGGACAAGAAGAGTACA	-23.0	-18.1	4.9
	Probe 2	GATACAGCTGGAAAAGAAGAGTAC	-22.0	-17.4	4.6
	Probe 3	TACAGCTGGACGAGAAGAGTAC	-22.7	-17.7	5.1

ΔG variation of free energy (kcal/mol) considering [Na^+] =0.15 M;
The underlined bases indicate the target SNVs.

(b)			Folding nt / ΔG	Hybridization nt / ΔG		
	Oligo	Sequence (5'-3')		T-DNA	Link	H1
KRAS gene	Link	GACGATACAGCTAATTCAGA-<br>GAGATCCGAATGCGCGTACT	6 nt<br>-0.6	**20 nt**<br>**-19.0**	-	-
	H1	AATGCGCGTACTGATACA-<br>AGTACGCGCATTCGGATCTC	24 nt<br>-11.1	4 nt<br>-4.6	**21 nt**<br>**-23.0**	-
	H2	TGTATCAGTACGCGCATTCG-<br>GAGATCCGAATGCGCGTA	24 nt<br>-14.9	4 nt<br>-4.6	16 nt<br>-18.9	**20 nt**<br>**-20.9**
NRAS gene	Link	AATACATGAGGACAGGCGAA-<br>GAGATCCGAATGCGCGTACT	6 nt<br>-0.6	**20 nt**<br>**-20.3**	-	-
	H1	AATGCGCGTACTGATACA-<br>AGTACGCGCATTCGGATCTC	24 nt<br>-11.1	7 nt<br>-4.0	**21 nt**<br>**-23.0**	-
	H2	TGTATCAGTACGCGCATTCG-<br>GAGATCCGAATGCGCGTA	24 nt<br>-14.9	3 nt<br>-3.3	16 nt<br>-18.9	**20 nt**<br>**-20.9**

T-DNA: template region; nt: number of complementary nucleotides in the loop or double-strand structure.
DG variation of free energy (kcal/mol) considering [Na^+] =0.15 M.

Table S2. Sequences of the oligonucleotides used for the AS-HCR method.

	Sequence (5’-3’)	Length (nt)	%GC	Tm (ºC)
	KRAS gene (Kirsten rat sarcoma-2 viral oncogene, codon 12-13)			
Primer 1	CTGAATATAAACTTGTGGTAGTTG	24	33	49.7
Primer 2	CTCTATTGTTGGATCATATTCGT	23	35	50.4
Probe WT	[BtnTg]-T10-GTTGGAGCTG**G**TG**G**CGTAGGC	21	67	59.0
Probe p.G12C	[BtnTg]-T10-AGTTGGAGCTGTTGGCGTAGGC	21	57	57.1
Probe p.G12S	[BtnTg]-T10-AGTTGGAGCTGATGGCGTAGGC	21	57	56.8
Probe p.G12R	[BtnTg]-T10-AGTTGGAGCTGCTGGCGTAGGC	21	62	59.1
Probe p.G13D	[BtnTg]-T10-GTTGGAGCTGGTGACGTAGGC	21	62	56.8
	NRAS gene (neuroblastoma ras viral oncogene, codon 61)			
Primer 1	GTTTGTTGGACATACTGGATA	21	38	47.1
Primer 2	TTCGCCTGTCCTCATGTATTG	21	48	53.4
Probe WT	[BtnTg]-T10-ATACAGCTGGA**CA**AGAAGAGTACA	24	42	55.0
Probe p.Q61K	[BtnTg]-T10-GATACAGCTGGAAAAGAAGAGTAC	24	42	53.2
Probe p.Q61R	[BtnTg]-T10-TACAGCTGGACGAGAAGAGTAC	22	50	54.8
	Control (β-actin gene, *ACTB* gene)			
Primer 1	GCACCACACCTTCTACAATGAG	22	50	53.6
Primer 2	GGCCACCAGAAGAGGTAGC	19	63	53.3
Control probe	[BtnTg]-T10-AACCGCGAGAAGATGACCCAGATCA	25	52	60.8
	HCR amplification			
Link 1	GACGATACAGCTAATTCAGAGAGATCCGAATGCGCGTACT	40	48	70.2
Link 2	AATACATGAGGACAGGCGAAGAGATCCGAATGCGCGTACT	40	50	71.1
H1	[Dig]AATGCGCGTACTGATACAAGTACGCGCATTCGGATCTC	38	50	70.4
H2	[Dig]TGTATCAGTACGCGCATTCGGAGATCCGAATGCGCGTA	38	53	71.8

[Dig]: digoxigenin-labeled; [BtnTg]: biotinTEG-labeled; T10: tail of 10 thymine tails;
Tm: melting temperature calculated by the Nearest Neighbor model[9].
The underlined bases indicate the target SNVs.

2. HCR method setup

Fabrication of chips and probe immobilization. The hybridization slides (25 mm×75 mm) were prepared by immobilizing the allele-specific probes on planar polycarbonate chips (Makrolon, Germany). Briefly, 30 nL of each biotinylated probe (200 nM) in printing buffer (10 mg/L streptavidin, 50 mM carbonate buffer, pH 9.6) were spotted on chips in a microarray format. To this end, a noncontact printer (AD 1500 BioDot Inc., USA) was employed that worked at 90% relative humidity. The array layout was four probes per array and three replicates per probe for the general tests. For multiplexing assays, seven probes and three replicates per probe were employed. The array also contained positive and negative probes as the controls. The layout enabled the simultaneous analysis of 12 samples. The spotted chips were incubated for 16 h at 37 °C. Then they were washed with PBS-T, rinsed with water, and air-dried.

A bioaffinity strategy was selected for the probe immobilization on the polycarbonate chip given the excellent features reported by several authors.[4,5,9] Under the selected conditions, the biotin-functionalized probes were attached via streptavidin to the chip surface by optimizing the dispensed concentration (Figure S3a). The calculated density of the immobilization probe was about 0.5 pmol/cm^2, and binding was stable for at least 4 weeks.

Reagents. The concentration of each oligonucleotide also played an important role in HCR system performance. We varied concentrations of H1 and H2, remained at an equimolar ratio, to maximize the formation of long H1/H2 hybrids. The best response was obtained using 100 nM of the link and 500 nM of H1 and H2, which implies that the saturation of HCR product growth was controlled by the opening and bonding of hairpins.

The essential components in the HCR reaction were checked after removing one reagent in each reaction solution. For assay optimization purposes, SNR signals were used to compare the results. The one-factor ANOVA reported significant differences in spot signal (p-value of 6.13×10^{-9}). When all the components were present, the DNA target was hybridized with the link, and the link was able to open one hairpin (H1) after hybridization between complementary regions to expose a new single-stranded region which was able to open the other hairpin

(H2) via an unbiased strand-displacement interaction. Therefore, a long dsDNA polymer structure was formed and easily detected (SNR>30). When DNA was absent, the link was closed and could not open the stem structure of H1 (SNR<3). When the link or H1 was absent, non-unions between the target DNA and hairpins occurred, and the two hairpins were concomitant and stable in a closed conformation (SNR<3).

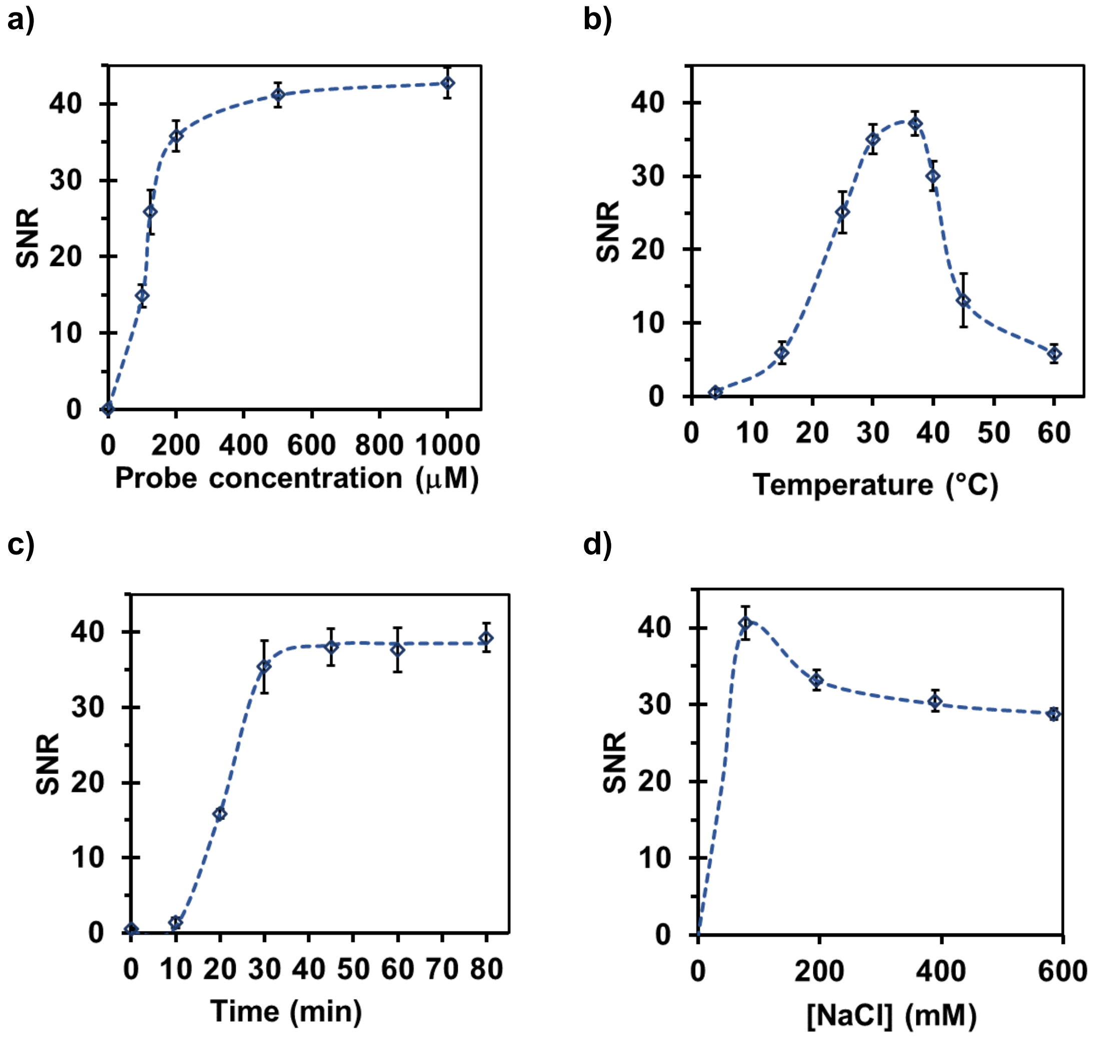

Figure S3. Evaluation of conventional HCR reaction conditions. a) probe b) Temperature. c) Time. d) Salt content in buffer solution. SNR: signal-to-noise ratio. Sample: wild-type *KRAS* template from cell culture (5 nM).

Only a detectable signal was obtained when DNA, link, and H1 were present, indicating a residual HCR process occurred (SNR>13). Although it was possible to bind the DNA/link hybrid to the stem structure of H1, no fully HCR-based amplification could be performed due to the unavailability of H2.

Incubation conditions. In order to enhance the signal, the incubation temperature, time, and hybridization buffer for the HCR method were studied. No response lower than 4°C was observed because H1 and H2 remained in a stable hairpin form. As the temperature rose, hairpins opened, which facilitated the union with the DNA/link hybrid, with a maximum of37°C (Figure S3b). Rapid amplification kinetics agrees with previous studies based on conventional HCR.[10] The highest spot intensity was achieved in 30 min (Figure S3c).

Regarding buffer composition, the NaCl concentration correlated with the registered signal (Figure S3d). The presence of salt ions (78 mM) reduced the electrostatic repulsion between the phosphate backbone of single-strand DNA and favored the formation of long double-strand DNA polymers. These results agree with the NaCl effect on hairpin folding and, consequently, on HCR efficiency[2]. The results from these experiments agree with the experimental conditions described for several HCR approaches for short templates.[11] No scientific studies were found in the literature that used HCR directly with genomic DNA.

Amplification factor estimation. Two model systems were designed for monitoring the recognition process but without HCR amplification (Table S3). Control 1 (or direct) consisted of two oligonucleotides called hybrid and marker, the latter of which was functionalized with the reporter molecule. The hybrid oligo sequence was complementary to the immobilized probe (3'-region) and the marker (5'-region). Control 2 (or hairpin-mediated) consisted of three oligonucleotides called hybrid, linker, and H1-marker, the last of which was functionalized with the reporter molecule. The hybrid oligo sequence was complementary to both the immobilized probe (3'-region) and the linker (5'-region). The linker sequence was also complementary to the hairpin structure.

In both controls, the stoichiometric ratio between the target molecule and the reporter molecule was 1:1, i.e., the systems worked as nonamplification controls of the biosensing process (Figure S4a). Linear transduction of the target hybridization was obtained until saturation because the response variation increased directly according to the target concentration (Figure S4b).

Table S3. Sequences of the oligonucleotides used as biosensing process controls.

		Sequence (5'-3')	Length (nt)	%GC	Tm (ºC)
Control 1	probe	[BtnTg]T10-GTTGGAGCTGGTGGCGTAGGC	31	67	67.3
	hybrid	GCTTCCTCTGTGTATTTGCCA-TTTTTT-CCTACGCCACCAGCTCCAAC	47	49	81.5
	marker	[Dig]-TGGCAAATACACAGAGGAAGC	21	48	59.5
Control 2	probe	[BtnTg]T10-GTTGGAGCTGGTGGCGTAGGC	31	67	67.3
	hybrid	TCTGAATTAGCTGTATCGTC-AAGGCACTCTT-GCCTACGCCACCAGCTCCAAC	52	52	84.5
	Link	GACGATACAGCTAATTCAGA-GAGATCCGAATGCGCGTACT	40	48	70.2
	H1-marker	[Dig]-AATGCGCGTACTGATACA-AGTACGCGCATTCGGATCTC	38	50	70.4

[Dig]: digoxigenin-labeled; [BtnTg]: biotin TEG-labeled; Tm: melting temperature.

The following experiments focused on estimating the amplification factor by the HCR method. The objective was to obtain the number of recognition/hybridization events between a pair of complementary DNA sequences that occurred in succession and yielded a long double strand hybrid. The assay results obtained by the HCR process were compared to the linear transduction of the target hybridization (Figure S4c). The signal intensity of the HCR products was 8.8-fold higher than the direct and hairpin-mediated approaches. This confirms that several reporter molecules were attached to each target DNA. Therefore, exponential HCR performance has a very high potential in the signal amplification of multiple targets.

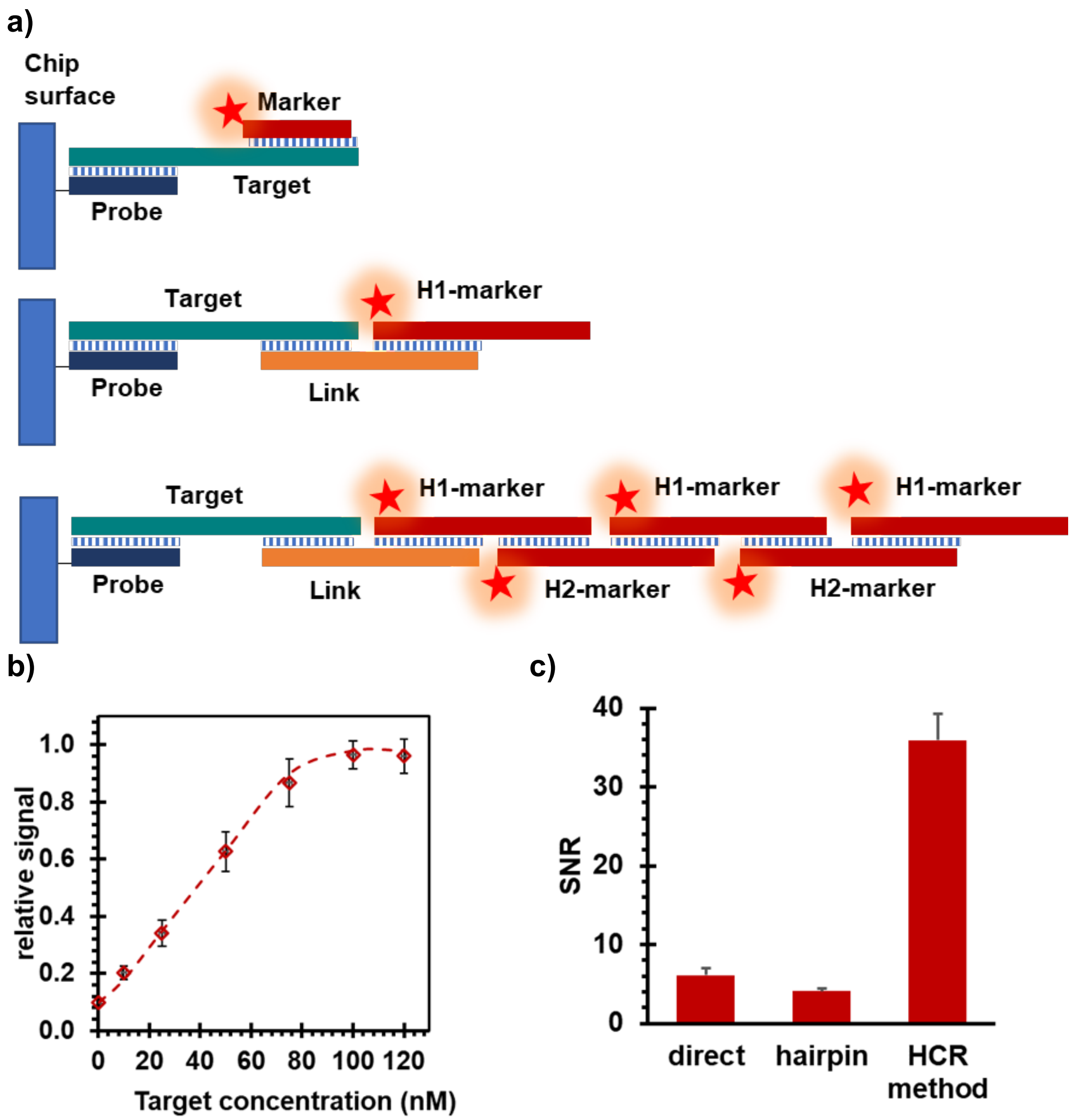

Figure S4. Linear vs. HCR approaches. a) Scheme of the tested biosensing methods: direct (top), hairpin-mediated (middle), and HCR (bottom). b) Effect of the control concentration for linear systems. c) Comparison of the spot intensities obtained for the studied biosensing approaches. SNR: signal-to-noise ratio.

3. Smartphone-based detection setup

Following the guidelines in the literature about point-of-care detection using smartphones,[4,12] a reading platform and methodology were designed and applied (Figure S5). The system can obtain a high-definition image of a conventional slide (25 mm×75 mm). A color pattern (grayscale) was added to the image array to correct variations in illumination.

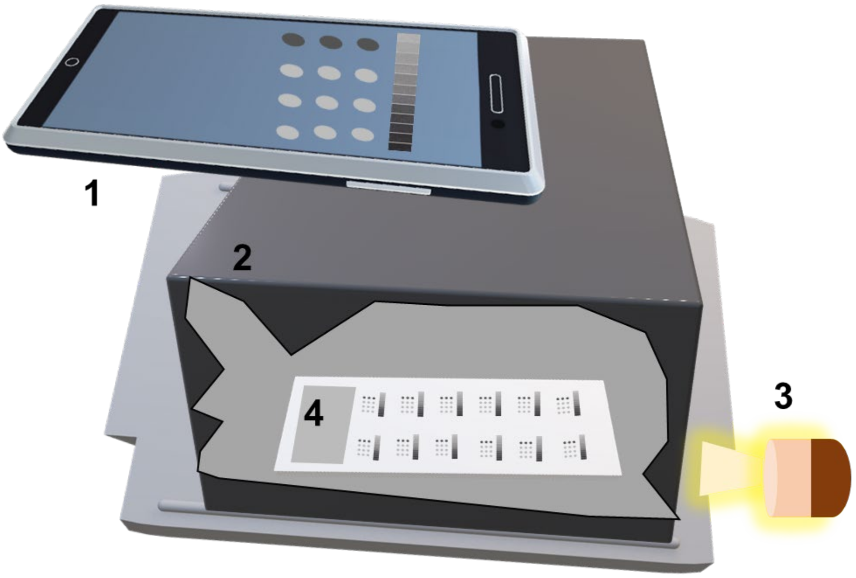

Figure S5. Assembly for the smartphone-based detection of HCR products. (1) Smartphone for detection (iPhone 11 Pro). (2) Homemade capture chamber (9×11.5×8 cm). The top layer had a 3×3 cm central window that fitted the protruding camera. The bottom was a translucent platform where the chip was located. (3) Illumination 5 W flash LED, e.g., smartphone torch. (4) Hybridization polycarbonate chip. A color palette is included and placed close to all the arrays to correct variations in illumination.

Several options for labelling nucleic acid samples in DNA microarray-based detection methods have been described.[13] Considering the assay requirements, HCR products were labelled using nanoparticles or digoxigenin for colorimetric detection (Figure S6).

(i) The direct detection of H1/H2 oligos labelled with gold nanoparticles (5 nm) led to unsatisfactory reflection-mode detection results (a low SNR). By including a silver developer reaction, enhanced detection was promoted because the reduction of silver ions to metallic silver generated an insoluble precipitate in only a few minutes (8 min). A significant attenuation, proportional to the analyte amount, was observed from the captured optical density on the positive spots.

(ii) The assay involved that labelled anti-digoxigenin antibody recognized digoxigenin-functionalized oligos. Later, two staining systems were compared: alkaline phosphatase combined with fast-red colorimetric substrate and gold nanoparticle combined with the silver developer. In both cases, an insoluble precipitate was obtained in the positive tests, which enabled accurate high-sensitivity optical detection. However, the nanoparticle system offered advantages for the enzyme conjugate by obtaining higher responses due to silver amplification, lower background signals, and a broad spectrum of use given its enzyme-free nature[4].

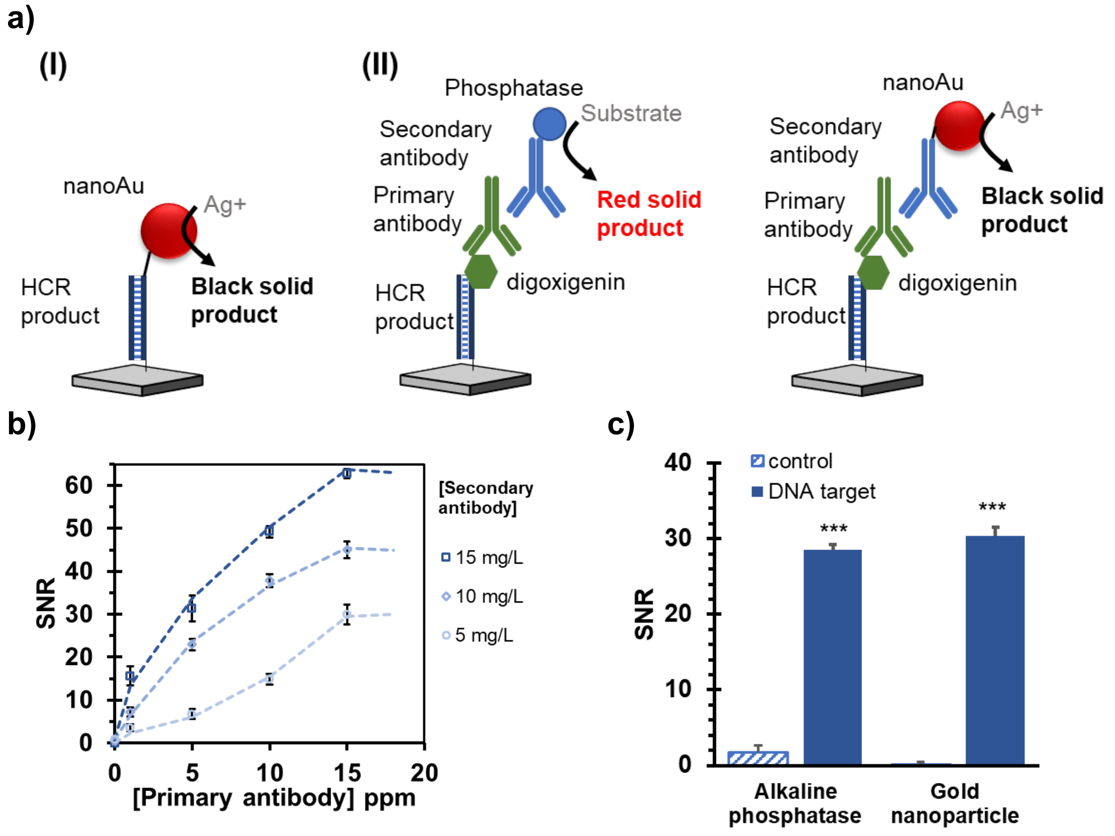

Figure S6. Optimization of chip immunostaining for smartphone-based detection in the colorimetric mode. a) Studied strategies: Oligonucleotides H1 and H2 were labeled at the 5'-end by 5-nm nanogold (i) or digoxigenin (ii) to generate a solid substrate. b) Effect of antibody concentrations. c) Comparison of developer agents for generating a colored solid deposit after dispensing the cocktail of antibodies.

Therefore, the selected staining of chips was an immunoassay. The working buffer was PBS-T, prepared as 10 mM of sodium phosphate, 0.15 M NaCl, and 0.05% Tween 20 at pH 7.4. The immunostaining solution consisted of an anti-digoxigenin rabbit antibody (Invitrogen, USA) at 5 ppm and a goat antirabbit-antibody labeled with gold (Abcam, UK) at 10 ppm diluted in PBS-T, and

incubated for 30 min. Silver enhancer solution, obtained from Sigma-Aldrich (USA), was incubated for 5 min.

To date, HCR products have been labeled mostly with electrochemical and fluorescent markers, which require specific instrumentation.[11,14,15] In the present study, proper integration into easy-to-use detection technologies took place, such as smartphones. This is an appealing HCR approach to run point-of-care tests.

In order to check chip imaging, a conventional documental scanner (Epson, USA) was used to record the array pattern. The scanner was connected to a personal computer via a universal device (USB 2.0). The image was digitized on the grayscale, 16-bits,1000 ppm, and the file was saved as TIFF using specialized software. Despite having a worse optical resolution than scanners, CMOS sensor chips embedded in phone cameras offer adequate imaging characteristics and wide availability, making them ideal detectors for cost-effective assays. Our results support the technical capabilities of smartphones as analytical readers for molecular diagnostic systems. High-resolution images were recorded and enabled the accurate, sensitive, and simple quantification of the spots generated based on a selective DNA assay (Figure S7). Thus smartphone-based detection is an attractive solution for low-cost, ubiquitous portable HCR applications.

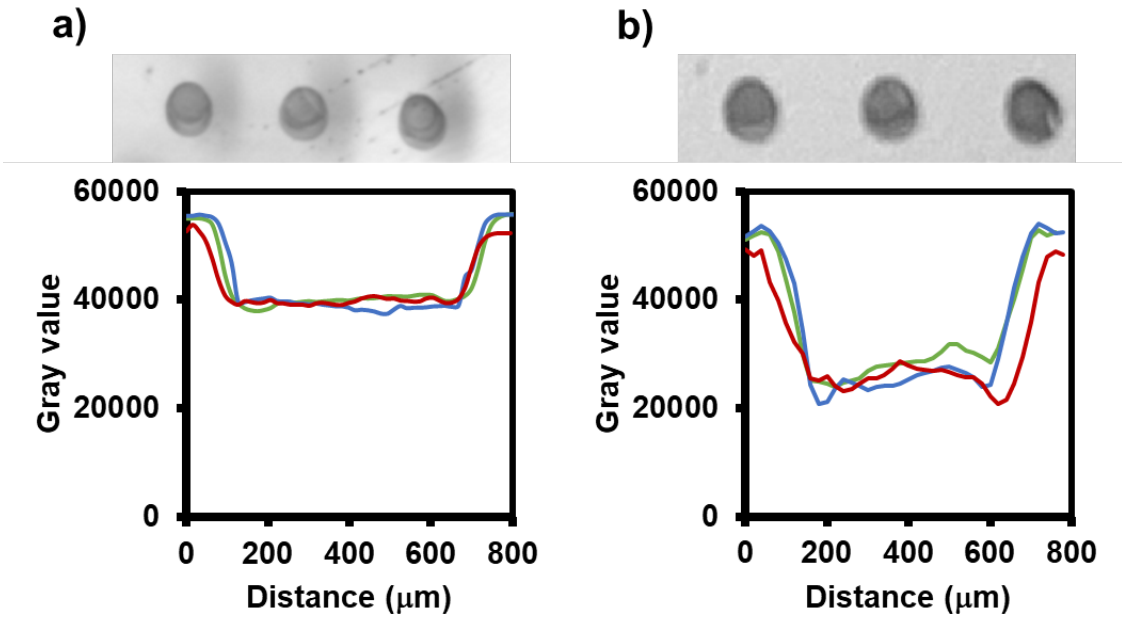

Figure S7. Assessment of the array quality obtained by a scanner (a) and a smartphone (b). (top) chip images; (bottom) cross-section profiles of spots. Software for the image analysis: Image J.

4. HCR method from RPA products

From the genomic DNA of human cell lines (SK-N-AS), target regions were amplified using specific primers and analyzed by gel electrophoresis. The separation of RPA products without purification was conducted in agarose gel 2% with SYBR safe diluted 1:10000 as a staining agent. For that, amplification solutions were mixed with loading buffer and transferred to gel wells. After separation at 90 V for 45 min, a gel image was captured (Figure S8a). The observed bands were 70 and 76 pb for *KRAS* and *NRAS* reactions, respectively. Thus, the results confirmed the correct amplification of target regions because the expected products were obtained. Based on the HCR method, RPA products were successfully detected (Figure S8b-c). The images captured by the smartphone showed a clear array pattern depending on the studied products. Also, spots intensities were high and reproducible.

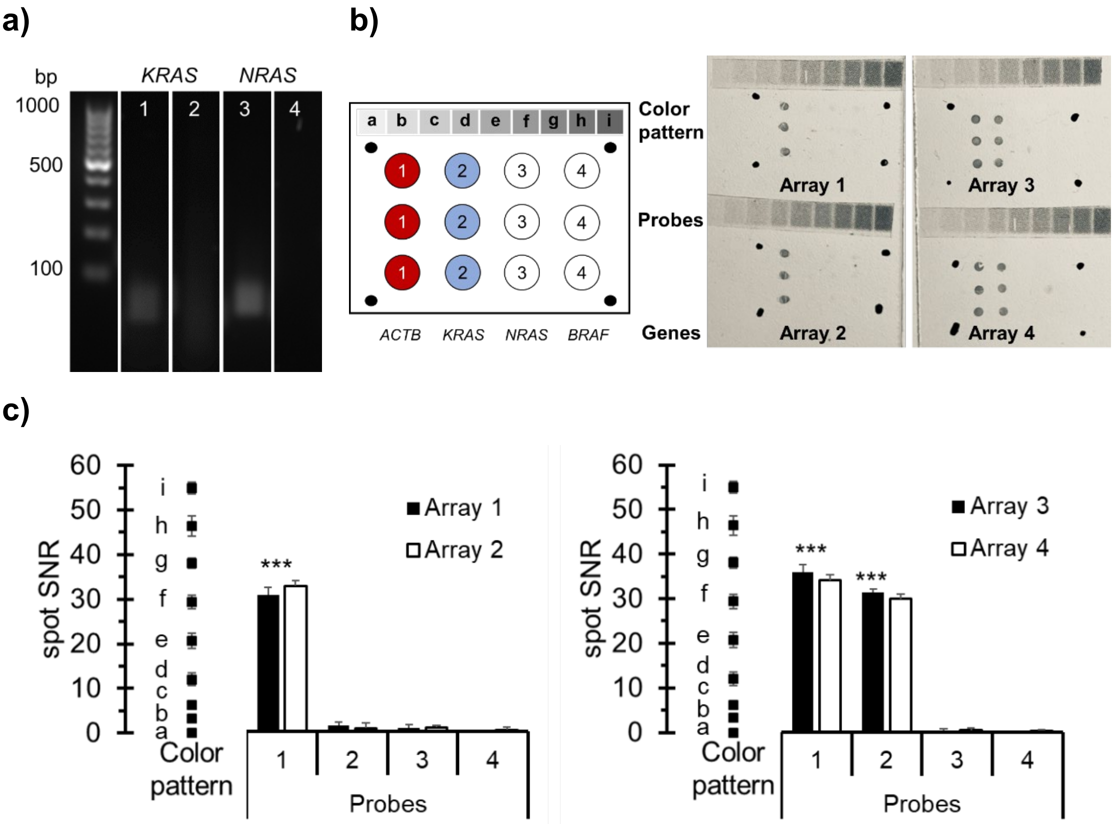

Figure S8. HCR method from RPA products. a) Agarose gel electrophoresis of RPA products using *KRAS* primers. Lanes 1 and 3: genomic DNA from human cells. Lanes 2 and 4: negative control. b) Qualitative results: Layout (left) and resulting images (right) taken by the smartphone using negative samples (arrays 1 and 2) and positive samples (arrays 3 and 4). Positional markers were put to delimit each array. b) Quantitative results: Spots signals arrays 1 and 2 (left) and arrays 3 and 4 (right). SNR: signal-to-noise ratio. *** Student's t-test p-values < 0.05. The color pattern ranges from a (lower intensity) to i (higher intensity).

5. Allele-specific HCR

PCR combined with allele-specific hybridization was used to compare the analytical performance of AS-HCR (Table S4). To do so, a reaction mixture was prepared with 1×DNA polymerase buffer, 3 mM of $MgCl_2$, 200 µM of each deoxynucleotide triphosphate, 300 nM of each primer, 4 ng of genomic DNA, and 1 unit of DNA polymerase (Biotools, Spain). For labeling purposes, 0.01 mM of digoxigenin-11-deoxyuridine triphosphate (Jena Bioscience, Germany) was added.

Table S4. Comparison of the PCR-chip and RPA-HCR methods

	**PCR-chip**	**RPA-HCR**
**Amplification**	PCR<br>Thermocycler<br>Labeled-dUTPs<br>35 cycles<br>(95ºC/57ºC/72ºC)<br>90 min	Fast RPA<br>Heater<br><br>37 ºC<br>10 min
	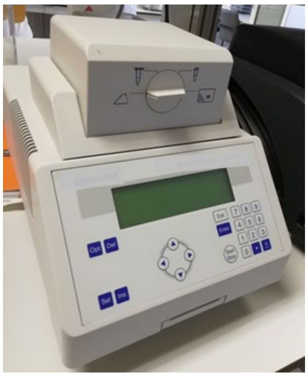	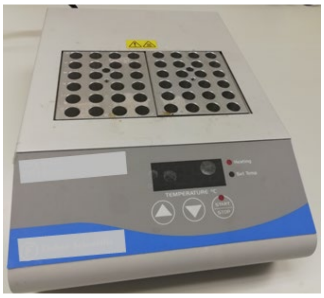
**Chip**	Polycarbonate 25×75 cm<br>Allele-specific probes	Polycarbonate 25×75 cm<br>Allele-specific probes
	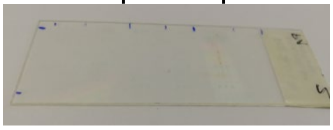	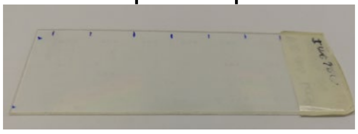
**Hybridization**	Restrictive conditions<br><br>37 ºC<br>60 min	Restrictive conditions<br>Reagents: Link, H1, H2<br>37 ºC<br>30 min
**Staining**	Colorimetric immunostaining	Colorimetric immunostaining
**Detector**	Smartphone	Smartphone

PCR was performed in a thermocycler (VWR, USA) under the following conditions: initial denaturation cycle of 95°C for 5 min, 35 denaturation cycles at 95°C for 30 s, annealing at 57°C for 30 s and elongation at 72°C for 60 s and, finally, one cycle extension at the end for 5 min. The amplified products (6 µL) were mixed with hybridization buffer (24 µL), heated (92°C, 10 min), and dispensed on sensing arrays. After incubation (37°C, 60 min), arrays were rinsed with washing buffer. Finally, colorimetric staining and detection were carried out following the same previously described procedure.

6. Application to clinical samples

Genomic DNA extraction. The genomic DNA of the cell lines was extracted using the PureLink Genomic DNA kit (Invitrogen, USA). The genomic DNA of the metastatic colorectal cancer samples was obtained with the QIAamp DNA Investigator Kit (Qiagen, Germany). DNA content was quantified in a NanoDrop 2000 spectrophotometer (Thermo Fisher Scientific, USA). A 260/280 nm absorbance ratio above 1.8 was considered to determine adequate purity.

Next-generation sequencing. Ion Torrent PGM technology (ThermoFisher Scientific, USA) was followed to validate the somatic mutations detection of patient samples. The Oncomine Solid Tumor DNA kit simultaneously analyzed hotspot mutations in 22 genes (including the *KRAS* and *NRAS* genes). A multiplex PCR amplification of 10 ng of genomic DNA generated the DNA barcoded libraries. The data from the sequencing runs were aligned to the hg19 human reference genome and variant calling.

AS-HCR method for single-nucleotide genotyping. The ability of the biosensing system to distinguish SNVs in real samples related to solid cancer screening was tested. As proof of concept, the proposed method was applied to identify the single-point mutations located at a specific locus of the *KRAS* gene (Genomic location 12: 25245347 (GRCh38)). Selective hybridization was achieved. Positive

signals (SNR>30) on the probes associated with the specific mutations were recorded for the mutant genomic DNA from cell cultures and patients. Negative or low signals (SNR<10) were obtained for the rest of the probes.

AS-HCR method for multiple targets. Chips contained 12 arrays for the simultaneous analysis of 12 samples. Each array was composed of the anchored probes, which were specific to the main intended mutations for the *KRAS* gene (codons 12 and 13) and *NRAS* gene (codon 61). The studied nucleotide changes were p.G12C (c.34G>T), p.G13D (c.38G>A), p.Q61K (c.181C>A) and p.Q61R (c.182A>G). The array layout was a square with positive controls in corners and three replicates for each target (Figure S9). The analyzed samples were obtained from patients with metastatic colon cancer (n=36) as a double-blind assay.

The AS-HCR results obtained from patient samples showed the genetic profile for the studied single-nucleotide variants, which provides valuable information about genes' mutational status. The capture images for the wild-type patients and mutant patients (p.G12C variant) are shown in the example. A clear discrimination pattern was observed, allowing the diagnosis, prognosis, and assignment of appropriate treatment. Hence, AS-HCR is suitable for centralized laboratories and point-of-care applications considering the employed resources.

Assay accuracy was validated by a parallel analysis of the patient samples following the reference method. Patients suffering from cancer and confirmed by their clinical history and other oncological tests were recruited, including cancer confirmation. A double-blind assay was performed, analyzing tissue samples. The novel method and genomic DNA sequencing results are shown in Table S5. Thus, the correct detection of single-nucleotide mutations was confirmed.

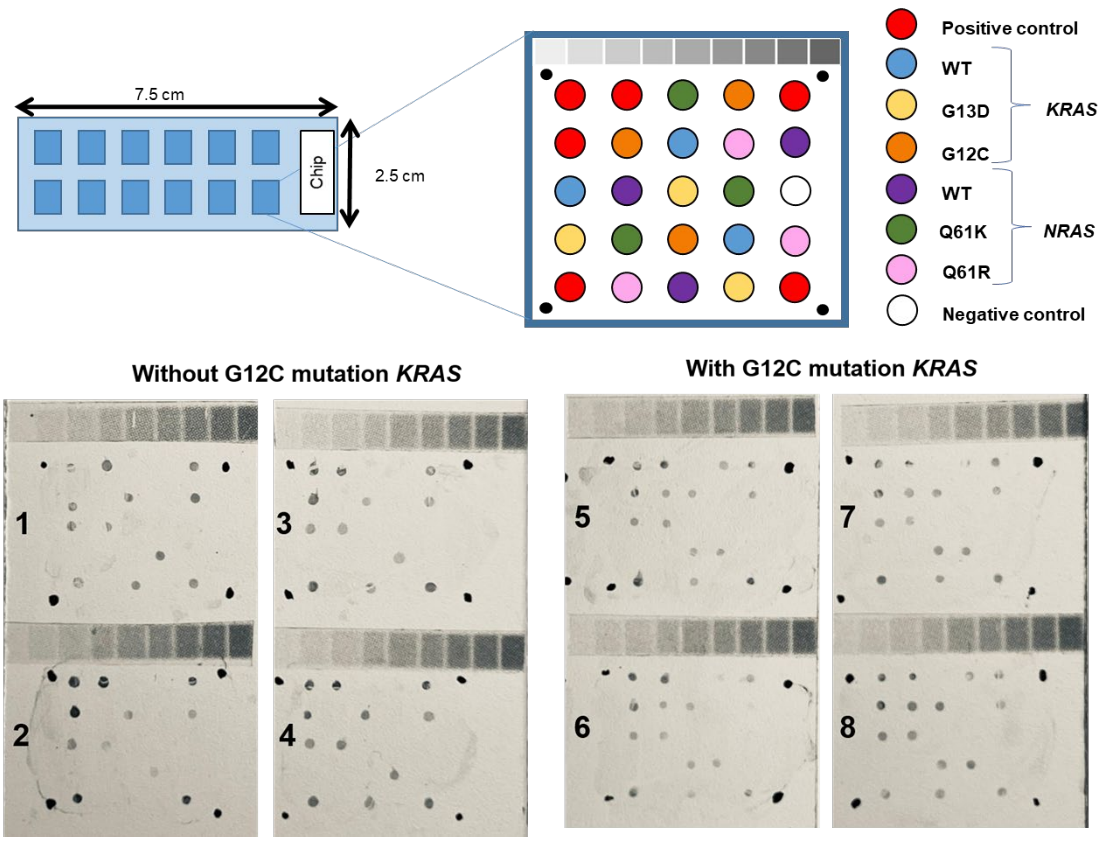

Figure S9. Images obtained from patient samples. In each chip, 12 samples were simultaneously analyzed by discriminating the presence of certain target SNVs.

Table S5. The mutation analysis of the clinical samples by PCR-based method and NGS technique for the target SNVs: *KRAS* gene (codon 12-13) and *NRAS* gene (codon 61).

Patient sample	*KRAS* Genotype	*NRAS* Genotype
1	WT	WT
2	WT	WT
3	WT	WT
4	WT	WT
5	WT	WT
6	WT	WT
7	WT	WT
8	WT	WT
9	WT	WT
10	WT	WT
11	WT	WT
12	WT	WT
13	WT	WT
14	WT	WT
15	WT	WT
16	WT	WT
17	WT	WT
18	WT	WT
19	WT	WT
20	WT	WT
21	WT	WT

Patient sample	*KRAS* Genotype	*NRAS* Genotype	DNA code	Protein code
22	M	WT	c.35G>A	p.G12D
23	M	WT	c.35G>A	p.G12D
24	M	WT	c.35G>A	p.G12D
25	M	WT	c.35G>A	p.G12D
26	M	WT	c.35G>T	p.G12V
27	M	WT	c.35G>T	p.G12V
28	M	WT	c.35G>T	p.G12V
29	M	WT	c.35G>C	p.G12A
30	WT	M	c.182A>T	p.Q61L
31	M	WT	c.34G>T	p.G12C
32	M	WT	c.34G>T	p.G12C
33	M	WT	c.34G>T	p.G12C
34	M	WT	c.34G>T	p.G12C
35	WT	M	c.181C>A	p.Q61K
36	WT	M	c.181C>A	p.Q61K

WT: wild-type
M: mutant

The developed HCR method is a powerful, cost-effective alternative for clinical applications and other scientific fields. In this study, we made several important research advances compared to other HCR-based methods (Table S6) or other methods for the genotyping of SNVs (Table S7). The advantages include being applied to complex clinical samples, ultrasensitive and selective genotyping of several SNVs, high-throughput analysis, excellent portability, and availability due to its compatibility with smartphone detection.

Table S6. Examples of different HCR sensing approaches classified according to the sensing principle.

Analytical platform	Labelled hairpins	Signal developer	Target	LOD*	Biological sample	Ref
**Fluorescence**						
Solution	Both with pyrene excimer	-	Target DNA	256 fM	Cell media	16
Solution	H1-H2 with poly-loop	Ag nanocluster	Let-7a miRNA	0.8 nM	No	17
Chip surface	4 hairpins with non-modification	SYBR Green I	Malaria 18sRNA and GAPDH mRNA	1 pM	Cell lysate	18
Au substrate	Both with azide at their ends	NIR fluorescent dyes	miRNAs-21	1 pM	Cell cultures	19
**Electrochemical**						
Au electrode	Non-modification	AgNPs	miRNA-17	2 aM	Cell lysates	20
ITO$^-$ electrode	Non-modification	Fc-PNA probes	Target DNA	100 fM	No	21
AuNP biocathode	Non-modification	$[Ru(NH_3)_6]^{3+}$	p53 gene fragment	20 aM	Cell lysate	22
**Electrochemiluminescence**						
Au electrode	Both with biotin at their ends	SA–AuNPs catalyze luminol	HIV-1 DNA fragment	5 fM	Artificial human serum	23
Au electrode	Non-modification	$[Ru(NH_3)_6]^{3+}$	E. coli DNA fragment	15 fM	No	24
**Colorimetric**						
Solution	Both with ssDNA sticky ends	AuNPs	Target DNA	50 pM	No	25
Solution	Non-modification	AuNPs	miR-10b, miR-21 and miR-141	20 fM	Cell line	26
Solution	H1 with biotin	Avidin-GOD mediated plasmonic triangular Ag nanoprism etching	p53 gene fragment	6 fM	No	27
Chip surface	H1 with G-quadruplex in the loop and in 1/3 of the stem	DNAzyme catalyze $ABTS^{2-}$oxidation after binding to hemin with H_2O_2.	Target DNA and sRNA.	7.5 nM	No	28
Solution	Both with 3/4 and 1/4 of the HRP-mimicking DNAzyme	Hemin/G-quadruplex nanowires catalyze $ABTS^{2-}$oxidation by H_2O_2	BRCA1 gene fragment	100 fM	No	29
Solution	Non-modification	Peroxidase mimics of Fe_3O_4 NPs and AuNPs	Y. pestis DNA fragment	100 pM	No	30
Solution	G-quadruplex structure in the stem of H1	Peroxidase activity by the binding between G-quadruplex and hemin	miR-21, miR-125b	5.6 nM	Diluted sample	31
Chip surface	H1-H2 with digoxigenin	Immunostaining. Smartphone detection	Multi-SNVs	100 fM	Patient tissues	Current paper

*__LOD:__ Limit of detection

Table S7. Currently available platforms and closely related SNV strategies for *RAS* genotyping.

Detection method	LOD[1]	Time	Multiplexing (samples/run)	Equipment	CE-IVD[2]	Comments	Ref.
**Sanger sequencing**	10-25 %	2 days	1-10	Sequencers	CRC RASseq	Requires a high amount of mutated DNA	32
**Pyrosequencing**	3–7 %	1 day	up to 96	Sequencers	Therascreen KRAS and NRAS Pyro	High error rate in homopolymer readout (>5 ntd)	33
**MALDI-TOF-MS**	5-10 %	4-5 hours	up to 96	Mass spectrometer	Agena Bioscience OncoCarta	Needs multiple preparation steps and high purity samples	34
**dHPLC**	3-5 %	Few hours	1	HPLC equipment	-	Cannot detect homozygous mutations directly nor determine the mutation type	35
**SNaPshot**	1-5 %	7 hours	10	Capillary genetic analyzer	SNaPshot Multiplex Kit	Multi-step process and each assay run separately	36
**PCR-RFLP**	5 %	4-5 hours	up to 96	Thermal cycler	-	Limit to targets containing sequences recognizable by the endonucleases	37
**Microarrays**	0.1-1 %	3 hours	hundreds	Chip reader	Randox biochip array	Low-cost. Challenging to optimize probes design and hybridization conditions	38
**HRM**	1-5 %	3 hours	up to 96	Real-time thermal cycler	LightMix NRAS ex2–4 KRAS ex4	Requires a previous PCR amplification step	39
**Real-time PCR and TaqMelt**	2.5-5 %	8 hours	up to 96	Specific real-time thermal cycler	COBAS 4800 KRAS assay	The activity of the probe influences sensitivity	40
**Scorpion ARMS**	1 %	8 hours	up to 96	Specific real-time thermal cycler	Therascreen KRAS	Scorpion probe design is very complex and laborious	41
**Blocked PCR**	0.1-1 %	2 hours	up to 96	Real-time thermal cycler	PNAClamp	Tedious blocker design	42
**AS-PCR**	0.1-1 %	3 hours	up to 96	Real-time thermal cycler	EnteroGen KRAS	May lead to false-positives results	43

(continue)

(continued)

**COLD-PCR**	0.01-1 %	< 3 hours	up to 96	Real-time thermal cycler	-	Requires precise control of working temperatures	44
**Droplet digital PCR**	0.001-0.1 %	2 hours	up to 96	Sophisticated thermal cycler	-	Absolute quantification. Costly for both the equipment and the reagents,	45
**Magnetic-assisted bioelectrocatalytic cycling**	0.005 %	2 hours	20	Chrono-amperemeter	-	Isothermal, low-cost. Multi-step protocol	46
**Electrochemical clamp assay**	1 fg·μL^{-1}	1 hour	23	Voltammeter	-	Isothermal, low-cost. Multi-step protocol	47
**SHERLOCKv2**	2 aM	2 hours	1-8	Fluorescence detector and lateral flow strips reader		Isothermal. Multi-step protocol. Limited access to reagents	48
**AS-HCR**	0.2 %	1 hour	12-100	Smartphone	-	Isothermal, low-cost, versatile transduction and POC detection. Two-step process RPA and detection of HCR products	Current paper

[1] LOD: Limit of detection expressed in percentage of mutated DNA respect to total DNA (%) or concentration (fg·μL^{-1} or aM), according to the results of each work.

[2] CE-IVD: In vitro diagnostic devices in European CE marking

Table S8. Potential applications of the AS-HCR and examples showing the clinical relevance of correct genotyping.

Diseases	Target gene	Target variation	Clinical sample	Examples
Cancer	KRAS<br>NRAS	multiple	Biopsy	49, 50
Retinitis pigmentosa	GARP2	Arg86Gln	Blood or buccal samples	51, 52
Hearing loss	GJB2<br>LRTOMT	Gly12Val<br>Trp77Arg<br>Arg81Gln	Blood or buccal samples	53, 54
Hepatitis C	RASSF 1A<br>CTLA-4<br>STAT4	rs2073498<br>rs5742909<br>rs7574865	Blood or buccal samples	55, 56
Pharmacogenetic applications	VKORC1<br>CYP2C9<br>CYP2C9	rs9923231<br>rs1799853<br>rs1057910	Blood or buccal samples	57, 58

7. References

(1) Dirks, R. M.; Pierce, N. A. Triggered amplification by hybridization chain reaction. *Proc. Natl. Acad. Sci.* **2004,** *101*, 15275-15278.
(2) Choi, H. M.; Beck, V. A.; Pierce, N. A. Next generation in situ hybridization chain reaction: higher gain, lower cost, greater durability. *ACS Nano*. **2014,** *8* (5), 4284-4294.
(3) Figg, C. A.; Winegar, P. H.; Hayes, O. G.; Mirkin, C. A. Controlling the DNA hybridization chain reaction. *J. Am. Chem. Soc.* **2020,** *142*, 8596-8601.
(4) Yamanaka, E. S.; Tortajada-Genaro, L. A.; Maquieira, Á. Low-cost genotyping method based on allele-specific recombinase polymerase amplification and colorimetric microarray detection. *Microchim. Acta.* **2017,** *184*, 1453-1462.
(5) Tortajada-Genaro, L. A.; Puchades, R.; Maquieira, Á. Primer design for SNP genotyping based on allele-specific amplification—Application to organ transplantation pharmacogenomics. *J. Pharm. Biomed. Anal*. **2017,** *136*, 14-21.
(6) Martorell, S.; Palanca, S.; Maquieira, Á.; Tortajada-Genaro, L. A. Blocked recombinase polymerase amplification for mutation analysis of PIK3CA gene. *Anal. Biochem.* **2018,** *544,* 49-56.
(7) Ang, Y. S.; Yung, L. Y. L. Rational design of hybridization chain reaction monomers for robust signal amplification. *Chem. Commun.* **2016,** *52*, 4219-4222.
(8) Markham, N. R.; Zuker, M. DINAMelt web server for nucleic acid melting prediction. *Nucleic Acids Res.* **2005,** *33*, W577-W581.
(9) Bumgarner, R. Overview of DNA microarrays: types, applications, and their future. *Curr. Protoc. Mol. Biol.* **2013,** *101*, 22-1.
(10) Li, F.; Tang, Y.; Traynor, S. M.; Li, X. F.; Le, X. C. Kinetics of proximity-induced intramolecular DNA strand displacement. *Anal. Chem*. **2016,** *88*, 8152-8157.
(11) Yang, D.; Tang, Y.; Miao, P. Hybridization chain reaction directed DNA superstructures assembly for biosensing applications. *TrAC, Trends Anal. Chem*. **2017,** *94,* 1-13.
(12) Kanchi, S.; Sabela, M. I.; Mdluli, P. S.; Bisetty, K. Smartphone based bioanalytical and diagnosis applications: A review. *Biosens. Bioelectron.* **2018,** *102,* 136-149.
(13) Gibriel, A.A. Options available for labelling nucleic acid samples in DNA microarray-based detection methods. *Brief. Funct. Genomics*, **2012**, *11*, 311-318.
(14) Zhang, C.; Chen, J.; Sun, R.; Huang, Z.; Luo, Z.; Zhou, C.; Wu, M.; Duan Y …Li, Y. The recent development of hybridization chain reaction strategies in biosensors. *ACS Sens.* **2020,** *5*, 2977-3000.
(15) Kramer, E. E.; Steadman, P. E.; Epp, J. R.; Frankland, P. W.; Josselyn, S. A. Assessing individual neuronal activity across the intact brain: using hybridization chain reaction (HCR) to detect arc mRNA localized to the nucleus in volumes of cleared brain tissue. *Curr. Protoc. Neurosci.* **2018**, *84*, e49.
(16) Huang, J.; Wu, Y.; Chen, Y.; Zhu, Z.; Yang, X.; Yang, C.J.; Wang, K.; Tan W. Pyrene-excimer probes based on the hybridization chain reaction for the detection of nucleic acids in complex biological fluids. *Angew. Chem., Int. Ed*. **2011**, *50*, 401–404.
(17) Qiu, X.; Wang, P.; Cao, Z. Hybridization chain reaction modulated DNA-hosted silver nanoclusters for fluorescent identification of single nucleotide polymorphisms in the let-7 miRNA family. *Biosens Bioelectron.* **2014**; *60*, 351–357.
(18) Xu, Y.; Zheng, Z. Direct RNA detection without nucleic acid purification and PCR: Combining sandwich hybridization with signal amplification based on branched hybridization chain reaction. *Biosens Bioelectron.* **2016**, *79*, 593–599.
(19) Yin, F.; Liu, H.; Li, Q.; Gao, X.; Yin, Y.; Liu, D. Trace MicroRNA Quantification by Means of Plasmon-Enhanced Hybridization Chain Reaction. *Anal Chem.* **2016**, *88*, 4600–4604.
(20) Miao, P.; Tang, Y.; Yin, J. MicroRNA detection based on analyte triggered nanoparticle localization on a tetrahedral DNA modified electrode followed by hybridization chain reaction dual amplification. *Chem Commun.* **2015**, *51*, 15629–15632.
(21) Xuan, F.; Fan, T. W.; Hsing, I. M. Electrochemical interrogation of kinetically-controlled dendritic DNA/PNA assembly for immobilization-free and enzyme-free nucleic acids sensing. *ACS Nano.* **2015**, *9*, 5027–5033.
(22) Gu, C.; Kong, X.; Liu, X.; Gai, P.; Li, F. Enzymatic Biofuel-Cell-Based Self-Powered Biosensor Integrated with DNA Amplification Strategy for Ultrasensitive Detection of Single-Nucleotide Polymorphism. *Anal Chem.* **2019**, *66*, 37–9.

(23) Wang, X.; Ge, L.; Yu, Y.; Dong, S.; Li, F. Highly sensitive electrogenerated chemiluminescence biosensor based on hybridization chain reaction and amplification of gold nanoparticles for DNA detection. *Sens. Actuators, B Chem.* **2015**, *220*, 942–948.
(24) Chen, Y.; Xu, J.; Su, J.; Xiang, Y.; Yuan, R.; Chai, Y. In Situ Hybridization Chain Reaction Amplification for Universal and Highly Sensitive Electrochemiluminescent Detection of DNA. *Anal. Chem.* **2012**, *84*, 7750-7755.
(25) Liu, P.; Yang, X.; Sun, S.; Wang, Q.; Wang, K.; Huang, J.; Liu, J.; He, L. Enzyme-free colorimetric detection of DNA by using gold nanoparticles and hybridization chain reaction amplification. *Anal Chem.* **2013**, *85*, 7689–7695.
(26) Rana, M.; Balcioglu, M.; Kovach, M.; Hizir, M.S.; Robertson, N.M.; Khan I.; Yigit, M.V. Reprogrammable multiplexed detection of circulating oncomiRs using hybridization chain reaction. *Chem. Commun.* **2016**, *52*, 3524–3527.
(27) Yang, X.; Yu, Y.; Gao, Z. A highly sensitive plasmonic DNA assay based on triangular silver nanoprism etching. *ACS Nano.* **2014**, *8*, 4902–4907.
(28) Dong, J.; Cui, X.; Deng, Y.; Tang, Z. Amplified detection of nucleic acid by G-quadruplex based hybridization chain reaction. *Biosens Bioelectron.* **2012**, *38*, 258–263.
(29) Shimron, S.; Wang, F.; Orbach, R.; Willner, I. Amplified detection of DNA through the enzyme-free autonomous assembly of hemin/G-quadruplex DNAzyme nanowires. *Anal Chem.* **2012**, *84*, 1042–1048.
(30) Zeng, C.; Lu, N.; Wen, Y.; Liu, G.; Zhang, R.; Zhang, J.; Wang, F.; Liu, X.; Li, Q.; Tang, Z.; Zhang, M. Engineering Nanozymes using DNA for catalytic regulation. *ACS Appl Mater Interfaces.* **2019**, *11*, 1790–1799.
(31) Park, C.R.; Rhee, W.J.; Kim, K.W.; Hwang, B.H. Colorimetric biosensor using dual-amplification of enzyme-free reaction through universal hybridization chain reaction system. *Biotechnol. Bioeng.* **2019**, *116*, 1567–1574.
(32) El Bairi, K. Illuminating Colorectal Cancer Genomics by Next-Generation Sequencing. Springer Nature Switzerland. **2020**.
(33) Araujo, L. H.; Souza, B. M.; Leite, L. R.; Parma, S. A.; Lopes, N. P.; Malta, F. S.; Freire, M. C. Molecular profile of KRAS G12C-mutant colorectal and non-small-cell lung cancer. *BMC Cancer*. **2021**, *21* (1), 1-8.
(34) Sherwood, J. L.; Müller, S.; Orr, M. C.; Ratcliffe, M. J.; Walker, J. Panel based MALDI-TOF tumour profiling is a sensitive method for detecting mutations in clinical non small cell lung cancer tumour. *PLoS One*. **2014**, *9* (6), e100566.
(35) Norman, R. L.; Singh, R.; Langridge, J. I.; Ng, L. L.; Jones, D. J. The measurement of KRAS G12 mutants using multiplexed selected reaction monitoring and ion mobility mass spectrometry. *Rapid Commun. Mass Spectrom.* **2020**, *34*, e8657.
(36) Penson, R. T.; Sales, E.; Sullivan, L.; Borger, D. R.; Krasner, C. N.; Goodman, A. K.; Growdon, W. B.; Schorge, J. O.; Boruta, D. M.; Birrer, M. J. A SNaPshot of potentially personalized care: Molecular diagnostics in gynecologic cancer. *Gynecol. Oncol*. **2016**, *141* (1), 108-112.
(37) Li, W. M.; Hu, T. T.; Zhou, L. L.; Feng, Y. M.; Wang, Y. Y.; Fang, J. Highly sensitive detection of the PIK3CA H1047R mutation in colorectal cancer using a novel PCR-RFLP method. *BMC cancer.* **2016**, *16* (1), 1-11.
(38) Damin, F.; Galbiati, S.; Soriani, N.; Burgio, V.; Ronzoni, M.; Ferrari, M.; Chiari, M. Analysis of KRAS, NRAS and BRAF mutational profile by combination of in-tube hybridization and universal tag-microarray in tumor tissue and plasma of colorectal cancer patients. *PloS One*. **2018**, *13* (12), e0207876.
(39) Suhaimi, N. A. M.; Foong, Y. M.; San Lee, D. Y.; Phyo, W. M.; Cima, I.; Lee, E. X. W.; Lim, W. Y.; Chia, K. S.; Likong, S.; Gong, M.; Lim, B.; Hillmer, A. M.; Koh, P. K.; Ying, J. Y.; Tan, M. H. Non-invasive sensitive detection of KRAS and BRAF mutation in circulating tumor cells of colorectal cancer patients. *Mol.Oncol.* **2015**, *9* (4), 850-860.
(40) Botezatu, I. V.; Nechaeva, I. O.; Stroganova, A. M.; Senderovich, A. I.; Kondratova, V. N.; Shelepov, V. P.; Lichtenstein, A. V. Optimization of melting analysis with TaqMan probes for detection of KRAS, NRAS, and BRAF mutations. *Anal. Biochem*. **2015**, *491*, 75-83.
(41) Matsunaga, M.; Kaneta, T.; Miwa, K.; Ichikawa, W.; Fujita, K. I.; Nagashima, F.; Furuse, J.; Kage, M.; Akagi, Y.; Sasaki, Y. A comparison of four methods for detecting KRAS mutations in formalin-fixed specimens from metastatic colorectal cancer patients. *Oncol. Lett.* **2016**, *12* (1), 150-156.

(42) Lázaro, A.; Tortajada-Genaro, L. A.; Maquieira, Á. Enhanced asymmetric blocked qPCR method for affordable detection of point mutations in KRAS oncogene. *Anal. Bioanal. Chem*. **2021**, *413* (11), 2961-2969.

(43) Barbano, R.; Pasculli, B.; Coco, M.; Fontana, A.; Copetti, M.; Rendina, M.; Vanna, M. V.; Graziano, P.; Maiello, E.; Fazio, V. M.; Parrella, P. Competitive allele-specific TaqMan PCR (Cast-PCR) is a sensitive, specific and fast method for BRAF V600 mutation detection in Melanoma patients. *Sci. Rep*. **2015**, *5* (1), 1-11.

(44) Galbiati, S.; Damin, F.; Burgio, V.; Brisci, A.; Soriani, N.; Belcastro, B.; Resta, C-D.; Gianni, L.; Chiari, M.; Ronzoni, M.; Ferrari, M. Evaluation of three advanced methodologies, COLD-PCR, microarray and ddPCR, for identifying the mutational status by liquid biopsies in metastatic colorectal cancer patients. *Clin.Chim. Acta*. **2019**, *489*, 136-143.

(45) McEvoy, A. C.; Wood, B. A.; Ardakani, N. M.; Pereira, M. R.; Pearce, R.; Cowell, L.; Robinson, C.; Lacopetta, F. G.; Spicer, A. J.; Amanuel, B.; Ziman, M.; Gray, E. S. Droplet digital PCR for mutation detection in formalin-fixed, paraffin-embedded melanoma tissues: a comparison with sanger sequencing and pyrosequencing. *J. Mol. Diagn.* **2018**, *20* (2), 240-252.

(46) Koo, K. M.; Trau, M. Direct enhanced detection of multiple circulating tumor DNA variants in unprocessed plasma by magnetic-assisted bioelectrocatalytic cycling. *ACS Sens*. **2020**, *5* (10), 3217-3225.

(47) Das, J.; Ivanov, I.; Montermini, L.; Rak, J.; Sargent, E. H.; Kelley, S. O. An Electrochemical Clamp Assay for Direct, Rapid Analysis of Circulating Nucleic Acids in Serum. *Nat. Chem.* **2015**, *7*, 569-575.

(48) Gootenberg, J. S.; Abudayyeh, O. O.; Kellner, M. J.; Joung, J.; Collins, J. J.; Zhang, F. Multiplexed and Portable Nucleic Acid Detection Platform with Cas13, Cas12a, and Csm6. *Science.* **2018**, *360*, 439-444.

(49) Yu, R. T. D.; Garcia, R. L. NRAS mutant E132K identified in young-onset sporadic colorectal cancer and the canonical mutants G12D and Q61K affect distinct oncogenic phenotypes. *Sci. Rep.* **2020**, *10* (1), 1-13.

(50) Daver, N.; Schlenk, R. F.; Russell, N. H.; Levis, M. J. Targeting FLT3 mutations in AML: review of current knowledge and evidence. *Leukemia*. **2019**, *33* (2), 299-312.

(51) Gibriel, A. A.; Tate, R. J.; Yu, Y.; Rawson-Lax, E.; Hammer, H. M.; Tettey, J. N.; Pyne, N. J.; Converse, C. A. The p. Arg86Gln change in GARP2 (glutamic acid-rich protein-2) is a common West African-related polymorphism. *Gene*. **2013**, *515*(1), 155-158.

(52) Daiger, S. P.; Sullivan, L. S.; Bowne, S. J. Genes and mutations causing retinitis pigmentosa. *Clin. Genet.* **2013**, *84*(2), 132-141.

(53) Gibriel, A. A.; Abou-Elew, M. H.; Masmoudi, S. Analysis of p. Gly12Valfs* 2, p. Trp24* and p. Trp77Arg mutations in GJB2 and p. Arg81Gln variant in LRTOMT among non syndromic hearing loss Egyptian patients: implications for genetic diagnosis. *Mol. Biol. Rep.* **2019**, *46*(2), 2139-2145.

(54) Sliwinska-Kowalska, M.; Pawelczyk, M. Contribution of genetic factors to noise-induced hearing loss: a human studies review. *Mutat. Res. - Rev. Mutat. Res.* **2013**, *752*(1), 61-65.

(55) Ali, N. A.; Hamdy, N. M.; Gibriel, A. A.; Mesallamy, H. O. E. Investigation of the relationship between CTLA4 and the tumor suppressor RASSF1A and the possible mediating role of STAT4 in a cohort of Egyptian patients infected with hepatitis C virus with and without hepatocellular carcinoma. *Arch. Virol.* **2021**, *166*(6), 1643-1651.

(56) Coppola, N.; Minichini, C.; Starace, M.; Sagnelli, C.; Sagnelli, E. Clinical impact of the hepatitis C virus mutations in the era of directly acting antivirals. *J. Med. Virol.* **2016**, *88*(10), 1659-1671.

(57) Lazaro, A.; Yamanaka, E. S.; Maquieira, A.; Tortajada-Genaro, L. A. Allele-specific ligation and recombinase polymerase amplification for the detection of single nucleotide polymorphisms. *Sens. Actuators B Chem.* **2019**, *298*, 126877.

(58) Rost, S.; Fregin, A.; Ivaskevicius, V.; Conzelmann, E.; Hörtnagel, K.; Pelz, H. J.; Lappegard, K.; Seifried,E.; Scharrer, I.; Tuddenham, E. G. D.; Müller, C. R.; Strom T. M.; Oldenburg, J. Mutations in VKORC1 cause warfarin resistance and multiple coagulation factor deficiency type 2. *Nature*, **2004**, *427*(6974), 537-541.
